# CHIP ubiquitylates NOXA and induces its lysosomal degradation in response to DNA damage

**DOI:** 10.1038/s41419-020-02923-x

**Published:** 2020-09-10

**Authors:** Marie-Christine Albert, Kerstin Brinkmann, Wojciech Pokrzywa, Saskia Diana Günther, Martin Krönke, Thorsten Hoppe, Hamid Kashkar

**Affiliations:** 1 Institute for Medical Microbiology, Immunology and Hygiene (IMMIH), Faculty of Medicine and University Hospital of Cologne, University of Cologne, Cologne, Germany; 2 Cologne Excellence Cluster on Cellular Stress Responses in Aging-Associated Diseases (CECAD), University of Cologne, Cologne, Germany; 3 The Walter & Eliza Hall Institute of Medical Research (WEHI) and Department of Medical Biology, University of Melbourne, Melbourne, VIC Australia; 4 Institute for Genetics, University of Cologne, Cologne, Germany; 5 Laboratory of Protein Metabolism in Development and Aging, International Institute of Molecular and Cell Biology, Warsaw, Poland; 6 Center for Molecular Medicine Cologne (CMMC), Faculty of Medicine and University Hospital of Cologne, Cologne, Germany

**Keywords:** Cell signalling, Post-translational modifications

## Abstract

The BH3-only protein NOXA is a regulator of mitochondrial apoptosis by specifically antagonizing the anti-apoptotic protein MCL-1. Here we show that the E3 ubiquitin ligase CHIP controls NOXA stability after DNA damage. Our findings reveal that CHIP and MCL-1 are binding partners of NOXA and differentially define the fate of NOXA. Whereas NOXA is initially targeted to mitochondria upon MCL-1-binding, CHIP mediates ubiquitylation of cytosolic NOXA and promotes lysosomal degradation of NOXA, which is not bound by MCL-1. Our data indicate that MCL-1 defines NOXA abundance and its pro-apoptotic activity. Increased NOXA levels beyond this threshold are effectively removed by lysosomal protein degradation triggered via CHIP-mediated ubiquitylation. Together, these results shed new light on regulatory circuits controlling DNA damage response and identified the E3 ligase CHIP as a new molecular guardian, which restricts the cytosolic accumulation of NOXA upon genotoxic stress.

## Introduction

The BH3-only protein NOXA is a p53-responsive gene involved in mitochondrial apoptosis triggered by DNA damage^1^. NOXA is critical in apoptosis due to its distinct capability to bind and antagonize MCL-1 (myeloid leukemia cell 1) an anti-apoptotic member of the BCL-2 protein family^2–5^. In contrast to other BH3-only proteins such as BIM, BID, and PUMA that interact with all anti-apoptotic BCL-2 family members (promiscuous binder), NOXA selectively binds only to a subset of BCL-2 proteins such as MCL-1 and A1 with high affinity (selective binder). Importantly, the “promiscuous” binders are potent killers as they can neutralize several anti-apoptotic BCL-2 proteins or directly activate the pore-forming proteins BAX and BAK. Selective binders like NOXA that do not target BAX or BAK promote cytotoxicity only in cooperation with a further complementary BH3-only member^3^.

Based on the prominent role of MCL-1 in human cancer^6,7^, previous studies focussed on NOXA mainly to address its role as a target for cancer therapy^8–12^. In particular, the use of proteasome inhibitors in tumor cells has been frequently associated with the accumulation of NOXA at the protein level, which in turn potentiates susceptibility towards cancer therapeutics by antagonizing MCL-1^9,13–15^. These data indicated that the cancer-associated modification of ubiquitin–proteasome system (UPS) leads to an increased NOXA ubiquitylation and proteasomal degradation^14,16^. Independent recent studies additionally showed that NOXA can promote MCL-1 degradation upon binding and involving the mitochondria-associated E3 ligase MARCH5^17–19^. MARCH5 is a component of mitochondrial quality control, which requires the mitochondrial outer membrane protein MTCH2 to mark MCL-1 for proteasomal degradation. This can only occur when MCL-1 is bound by NOXA and results in the turn-over of the mitochondrial MCL-1/NOXA protein complex^18^.

Ubiquitylation is a post-translational modification of substrate proteins with ubiquitin (Ub). It acts as a versatile cellular signal that modulates a wide range of biological processes including protein degradation, DNA repair, apoptosis, endocytosis, and inflammation^20,21^. Substrate ubiquitylation is achieved by the sequential cooperation of an Ub-activating enzyme (E1), an Ub-conjugating enzyme (E2), and an E3 Ub ligase that catalyzes the covalent attachment of Ub to the internal lysine residues of target proteins^22,23^. Modification of the N-terminus (M1) or one of the seven lysine residues (K6, K11, K27, K29, K33, K48, and K63) of the substrate-attached Ub leads to the formation of various polymeric chains (designated as “ubiquitin code”) adopting distinct conformations and conferring different outcomes for the ubiquitylated substrates^24^. The human genome encodes two E1s, approximately 38 E2s, and more than 600 E3s. Within this enzymatic cascade, E3s have a pivotal role in selecting substrates and, together with E2~Ub, they coordinate the conjugation of the specific Ub linkages^20^. The specificity of Ub signaling is achieved by alternative conjugation signals (mono-ubiquitylation and different ubiquitin chain linkages).

Here we delineate the molecular mechanisms that control NOXA protein abundance in cells under genotoxic stress. Our data identify CHIP (carboxyl-terminus of Hsc70-interacting protein) (also called STUB1, STIP1 homology and U-Box containing protein 1)^25,26^ as the critical E3 ligase that binds and ubiquitylates NOXA. CHIP-mediated ubiquitylation of NOXA subsequently promotes its lysosomal targeting and degradation. In addition to the previous knowledge about the UPS-dependent regulation of NOXA in cancer chemoresistance and mitochondrial quality control involving MARCH5, our findings here reveal another layer of ubiquitin-based regulation of NOXA protein abundance. We show that cytosolic NOXA protein, which is not bound to MCL-1 and expressed exceedingly beyond the threshold of MCL-1, is targeted for lysosomal degradation. This process represents a cellular surveillance mechanism that prevents the cytosolic accumulation of NOXA during DNA damage-induced cellular stress responses.

## Materials and methods

### Plasmids and siRNA

The generation of amino-terminal GFP-, HA-, or myc-tagged NOXA^wt^ and NOXA^K0^ has been described previously^14^. Oligonucleotides for PCR were obtained from Eurofins MWG Operon. NOXA^KxR^ and NOXA^Kxonly^ mutants have been generated by site-directed mutagenesis PCR using specific primers harboring the desired mutation with NOXA^wt^ or NOXA^K0^ cDNA from HeLa cells as a template. Phusion High Fidelity DNA Polymerase Mastermix (ThermoFisher Scientific) was used for PCR reaction. For the generation of GFP-tagged and mCherry-tagged proteins, the corresponding ORFs were amplified by PCR and cloned into pEGFP-C3 and pmCherry-C3 vector (purchased from Clontech-Takara Bio Europe), respectively. GFP- and mCherry-tagged Lamp-1 were generated by cloning of Lamp-1 ORF into pEGFP-N3 and pmCherry-N3, respectively. For the generation of HA-, myc-tagged and non-tagged proteins, the corresponding ORFs were amplified by PCR and cloned into a pcDNA-3.1+ vector (Invitrogen) containing amino-terminal HA-, myc-tag sequence, or no tag. Strep-ubiquitin constructs were a kind gift of Prof. Mads Gyrd-Hansen (Oxford, UK). HA-NOXA^3E^ construct was kindly provided by Prof. Eric Eldering (AMC, Netherlands). Flag-ubiquitin constructs were kindly provided by Prof. David Komander (WEHI, Australia) to clone ORF-containing ubiquitin mutants into Strep-vector. Restriction enzymes and ligase for digestion and ligation of constructs and inserts were purchased from ThermoFisher Scientific.

siRNAs *siSCR*, *siCHIP*#1, *siCHIP*#2, *siRBX1*#1, *RBX1*#2 were designed and purchased from Eurofins Genomics (sequences see below).

*siSCR*: Sense 5′-(GGA UUA CUU GAU AAC GCU AUU)TT-3′,

Antisense 5′-(AAU AGC GUU AUC AAG UAA UCC)TT-3′;

*siCHIP*#1: Sense 5′-(CCA CUA UCU GUG UAA UAA A)TT-3′,

Antisense 5′-(UUU AUU ACA CAG AUA GUG G)TT-3′;

*siCHIP*#2: Sense 5′-(GGG ACG ACA UCC CCA GCG CUC U)TT-3′,

Antisense 5′-(AGA GCG CUG GGG AUG UCG UCC C)TT-3′;

*siRBX1*#1: Sense 5′-(GAA GCG CUU UGA AGU GAA A)TT-3′

Antisense 5′-(UUU CAC UUC AAA GCG CUU C)TT-3′;

*siRBX1*#2: Sense 5′-(GCA UAG AAU GUC AAG CUA A)TT-3′,

Antisense 5′-(UUA GCU UGA CAU UCU AUG C)TT-3′

*siNOXA*: Sense 5′-(GCU GUG AUA ACG UGA AAC CTT)TT-3′

Antisense 5′-(AAG GUU UCA CGU UAU CAC AGC)TT-3′

### Cell culture, transfection, and drug treatment

HeLa, HEK293T, HCT, U2OS, and MCF7 cells were purchased from and authenticated by ATCC. HeLa, HEK293T, U2OS, and MCF7 cells were cultured in DMEM (Biochrom) and HCT cells were cultured in McCoy (Biochrom) supplemented with 10% FCS (Biowest) and 100 µg/ml streptomycin and 100 unit/ml penicillin (Biochrom) at 37 °C on plastic culture flasks (TPP) at saturated humidity and a CO_2_ saturation of 5%. Cells were routinely tested for mycoplasma contaminations by PCR.

For DNA damage induction, cells were treated with Doxorubicin (DOX, Sigma-Aldrich) or Etoposide (ETO, Sigma-Aldrich). Staurosporine (STS) and zVAD-fmk (zVAD) was purchased from Enzo Life Sciences.

For lysosomal inhibition, cells were treated with 100 nM Bafilomycin A1 (Biomol), 30 mM NH_4_Cl (Merck), or 10 µM Chloroquine (Sigma-Aldrich) for 16 h.

For proteasomal inhibition, cells were treated with 5 µM MG132 (Sigma-Aldrich) or 50 nM Bortezomib (Teva) for 16 h.

Lipofectamine^®^ LTX with Plus™ (Invitrogen) or Polyethylenimin (PEI) (Polysciences Europe GmbH) was used for plasmid transfection in HeLa and HEK293T cells according to the manufacturer’s instructions. For siRNA transfection, Lipofectamine^®^ RNAi MAX™ (Invitrogen) was utilized according to the manufacturer’s instructions.

### RNA extraction, reverse transcription, and qPCR

Total RNA was isolated from HeLa cells using the standard phenol-chloroform-method after resuspension in TRIzol^®^ (Ambion). cDNA was synthesized using RevertAid^TM^ Premium First Strand cDNA Synthesis Kit (ThermoFisher Scientific) using poly-dT-primers according to the manufacturer’s instructions. qPCR was performed with specific primers to amplify *Noxa* transcript (fw GCT CCA GCA GAG CTG GAA GT; rev CCA TCT TCC GTT TCC AAG GGC), *CHIP* (fw GTA TTA CAC CAA CCG GGC CT; rev ATT GGC GAT GGC CTC ATC AT) and *Actin* serving as reference (fw GGA GGA GCT GGA AGC AGC C; rev GCT GTG CTA CGT CGC CCT G) using LightCycler^®^ SYBR-Green I Mix (Roche Applied Sciences) with a 96-well-plate Multicolor Real-Time PCR Detection System (iQTM5, BIO-RAD). Samples were measured as independent biological replicates and values are normalized to *Actin*, log2 transformed (ΔΔCT), and presented as mean ± standard error of the mean (SEM).

### Microscopy

For confocal microscopic analyses, cells were incubated with 100 nM MitoTracker (ThermoFisher Scientific) in warm culture medium (DMEM) for 45 min in a CO_2_-incubator (5%, 37 °C). Nuclei were stained with 300 nM DAPI (ThermoFisher Scientific) diluted in washing buffer (0.1% Saponin (w/v) in PBS). Cells were fixed with 3% PFA in PBS immobilized on coverslips and incubated for 30 min in blocking buffer (0.1%, Saponin (w/v) 3%, bovine serum albumin (BSA) (w/v) in PBS). In a humidity chamber, cover slides were first incubated for 2 h at RT or at 4 °C overnight with primary antibodies diluted in blocking buffer. After incubation with the primary antibody, the cells were washed 3× with washing buffer for 10 min and incubated with the appropriate fluorochrome-conjugated secondary antibody for 1 h at RT. Cells were washed 3× with washing buffer and PBS before mounting them on glass slides with Mowiol mounting reagent.

For live-cell imaging, HeLa cells were transfected with the indicated constructs and incubated for 24 h. To visualize mitochondria, 100 nM MitoTracker (ThermoFisher Scientific) was added in a warm medium for 45 min in a CO_2_-incubator at 37 °C/ 5% CO_2_.

Confocal imaging of live and fixed cells was performed on a spinning disk confocal microscope UltraView VoX from Perkin Elmer (software Volocity). For live-cell imaging, an additional incubator at 37 °C, 5% CO_2_ was used to sustain normal conditions. Images represent extended focus overlays of several *z*-stacks.

Superresolution imaging of fixed samples was performed on a confocal superresolution microscope TCS SP8 gSTED 3× from Leica Microsystems (Software LAS X) equipped with a white light laser for excitation and a 592 nm laser for depletion. A ×93 glycerol objective with a numerical aperture of 1.3 (Leica Microsystems) was used.

All acquired images (confocal and STED) were deconvolved using the software Huygens Professional (Scientific Volume Imaging) and further processed using AdobePhotoshop™.

For electron microscopy (EM) cells were incubated in fixation buffer (2% glutaraldehyde, 2% sucrose in PBS, pH 7.4) for 30 min and the reaction was quenched with quenching buffer (100 mM KCN, 100 mM Glycin) for 90 min. After washing with PBS cells were incubated for 40 min in 50 mM NH_4_Cl and subsequently incubated for 40 min in 10 mg/ml NaBH_4_. Cells were washed 5 × 5 min in 50 mM Tris (pH 7.4) and incubated overnight at 4 °C. For contrasting, 1 ml of 1 mg/ml DAB (in Tris) was mixed with 3 µl 30% H_2_O_2_ and incubated for 30 min or 50 min. The reaction was stopped by the addition of 50 mM Tris (pH 7.4) and washed with Tris 3 × 5 min. Next, cells were incubated for 30 min at 4 °C in 1% OsO_4_ + 1.5% potassium ferrocyanide and washed 3 × 5 min with H_2_O_dest_. For dehydration, cells were incubated at 4 °C for 5 min in 50% EtOH, 5 min 70% EtOH, 10 min 90% EtOH, 3 × 5 min in 100% EtOH, 2 h in Epon (+EtOH 1:1) and left overnight in Epon. Samples were embedded in Epon and cured for 48 h at 60 °C. 70-nm-ultrathin sections were cut using the Leica UC7 Ultramicrotome. Images were acquired using a JEM2100Plus (Jeol) transmission electron microscope equipped with a Gatan OneView 4k camera.

### Antibodies for IF

#### Primary antibodies

Anti-Tomm20 (Abcam #ab56783-100); anti-NOXA (clone 114C307 Merck #OP180); anti-Sec23 (Abcam #ab87352); anti-Cop1β (Abcam #ab2899); anti-EEA1 (clone C45B10, Cell Signaling #3288P); anti-APPL (clone D83H4, Cell Signaling #38588); anti-Syntaxin6 (clone C34B2, Cell Signaling #2869P); anti-Vamp3 (Abcam); anti-GopC (Cell Signaling #4646P); anti-Rab5 (clone C8B1, Cell Signaling #3547P); anti-Rab7 (clone D95F2 XP (R), Cell Signaling #9367S); anti-Rab9 (clone Mab9, Enzo, #ALX-804-286-R100); anti-Lamp-1 (clone D2D11 XP (R), Cell Signaling #9091S).

#### Secondary antibodies

Rabbit-anti-mouse-Alexa594 (Invitrogen #A11062); goat-anti-mouse-Alexa488 (Invitrogen #A11001); rabbit-anti-mouse-Alexa633 (Invitrogen #A21063); chicken-anti-rabbit-Alexa594 (Invitrogen #A21442); goat-anti-rabbit-Alexa488 (Invitrogen #A11008); goat-anti-rabbit-Alexa633 (Invitrogen #A21070).

### Whole-cell lysate, immunoprecipitation, and streptavidin-pulldown

For whole-cell lysates, cells were pelleted (3 min, 700 × *g*) and washed 2× with chilled PBS. The cell pellet was resuspended in CHAPS lysis buffer (2× volume of pellet, 10 mM HEPES pH 7.4, 150 mM NaCl, 1% (w/v) CHAPS, and complete protease inhibitor cocktail (Roche)) and incubated on ice for 30 min. The lysate was centrifuged for 20 min at 20,800 × *g* and 4 °C.

For Strep-pulldown (PD) analysis the cell pellet was resuspended in Strep-lysis buffer (25 mM HEPES, 150 mM KCl, 2 mM MgCl_2_, 1 mM EGTA, 0.5% Triton X-100, 625 µg/ml), *N*-ethylmaleimide (NEM) (Sigma-Aldrich), 1× complete protease inhibitor cocktail (Roche) and incubated for 30 min on ice. Lysates were centrifuged for 20 min at 20,800 × *g* at 4 °C. Strep-Tactin^®^ Sepharose^®^ beads (IBA) were washed with Strep-lysis buffer prior to incubation with lysate. For each sample, 50 µl of beads were used and incubated at 4 °C for 3 h or overnight on a rotating wheel. After incubation, beads were washed 3× with washing buffer (0.05% Tween 20 in PBS) prior to addition of 50–70 µl 2× Laemmli sample buffer (5×: 0.6 M Tris-HCl pH 6.8, 144 mM SDS, 25% (v/v) glycerol, 0.1% (w/v) bromphenol blue, 5% (v/v) β-mercaptoethanol), and boiled for 5 min at 95 °C.

Immunoprecipitation of tagged proteins was performed with the µMACS Isolation Kit (HA, myc, GFP) according to the manufacturer’s instructions (Miltenyi Biotec). In brief, cell pellets were resuspended in lysis buffer (Miltenyi Biotec), incubated for 30 min on ice, and centrifuged for 20 min at 20,800 × *g* at 4 °C. Samples were washed with 1× lysis buffer, 2× wash buffer 1, and 1× wash buffer 2 for interaction studies. Samples were washed with 2× lysis buffer, 6× wash buffer 1, and 1× wash buffer 2 for ubiquitylation analysis.

### Endogenous IP

For immunoprecipitation of endogenous protein interactions, cells were washed twice with chilled PBS and centrifuged at 700×*g* for 3 min at 4 °C. Proteins were cross-linked with disuccinimidyl suberate (DSS, ThermoScientific) prior to lysis. In brief, 2.5 mM DSS in PBS was added to cells and incubated for 30 min at RT. The reaction was quenched by incubation with 1.25 M glycine in PBS for 15 min RT. The cell pellet was resuspended in lysis buffer (Miltenyi Biotec) and incubated for 30 min on ice and centrifuged for 20 min at 20,800 × *g* at 4 °C. Lysates were first incubated for 1 h with a respective antibody against NOXA (clone N-15, Santa Cruz #sc-26917; discontinued) at 4 °C on a rotating wheel followed by 1 h incubation of MACS Protein G beads (Miltenyi Biotec). IP was conducted according to the manufacturer’s instructions. Samples were washed with 1× lysis buffer, 2× wash buffer 1, and 1× wash buffer 2.

For immunoprecipitation of endogenous ubiquitylated NOXA, cells were washed twice with chilled PBS and centrifuged at 700 × *g* for 3 min at 4 °C. The cell pellet was resuspended in RIPA buffer (1% Triton, 150 mM NaCl, 50 mM Tris, 1% SDS, 0.5% deoxycholate) and incubated for 30 min on ice and centrifuged for 20 min at 20,800 × *g* at 4 °C. Prior to antibody incubation, SDS concentration was diluted to 0.1% SDS. Lysates were first incubated for 1 h with a respective antibody against NOXA anti-NOXA (clone 114C307, Merck #OP180) at 4 °C on a rotating wheel followed by overnight (16 h) incubation with Protein A/G agarose beads (Santa Cruz). Beads were washed 3× with PBST (PBS + 0.1% Tween 20).

### Control IgGs

Normal Mouse IgG (Santa Cruz #sc-2025), Normal Goat IgG (Santa Cruz #sc-2755).

### Nuclear extracts

To determine PARP cleavage, nuclear extracts were performed after cell lysis with CHAPS. Cell pellets containing cellular membranes as well as the nucleus were resuspended with 25 µl Urea-Laemmli buffer (Laemmli sample buffer (2×), 8 M urea, 6% β-mercaptoethanol) and incubated for 5 min at 95 °C. Next, nuclear lysates were diluted with 25 µl 2× Laemmli and incubated for 5 min at 95 °C^27^.

### Mass spectrometry (MS) IP

For MS NOXA-IP, HEK293T cells were transfected with HA-NOXA for 36 h and treated with 1 µM DOX for 16 h. After harvesting and washing with PBS, cells were cross-linked by incubation in 0.025% glutaraldehyde (GA)/PBS (w/v) for 10 min, 30 °C. The reaction was quenched by 100 mM Tris-HCl, pH 7.5 for 10–15 min at RT. The cell pellet was resuspended in Strep-lysis buffer, incubated for 30 min on ice, and centrifuged for 20 min at 20,800 × *g* at 4 °C. IP was conducted according to the manufacturer’s instructions with Protein G beads (Miltenyi Biotec) and NOXA-specific antibody (clone N-15, Santa Cruz #sc-26917; discontinued). Samples were washed with 1× Strep-lysis buffer, 2× wash buffer 1, and 1× wash buffer 2. Samples were eluted with 8 M urea.

### Antibodies and reagents for western blotting

#### Primary antibodies

Anti-β-Actin (clone AC-15, Sigma-Aldrich #A5441), anti-PARP (clone C2-10, BD Pharmingen^TM^ #556362), anti-HA (Abcam #ab9110), anti-myc (clone 9E10, Sigma-Aldrich #M4439), anti-NOXA (clone 114C307, Merck #OP180), anti-MCL-1 (clone D35A5, Cell Signaling #54535), anti-A1/BFL-1 (clone D1A1C, Cell Signaling #14093S) anti-RBX1 (clone D3J5I, Cell Signaling #11922S), anti-CHIP (clone C3B6, Cell Signaling #2080S).

#### Secondary antibodies

Anti-Mouse IgG HRP (Sigma-Aldrich #061M4792), anti-Rabbit IgG HRP (Cell Signaling #7074S).

For size reference, either Prestained PAGE ruler (Fisher Scientific) or Prestained PAGE ruler plus (Fig. 4c) (Fisher Scientific) was used.

### In vitro ubiquitylation assays

For in vitro ubiquitylation and binding assays recombinant proteins were purchased from Boston Biochem (ubiquitin, CHIP-6xHis, UbcH5a, UbcH5b, UBE1), Abcam (His-MCL-1), Origene (flag-NOXA), and BPS Bioscience (flag-XIAP)^28^. Recombinant protein NOXA^wt^ was synthesized by Lifetein (Somerset, USA) (Sequence NOXA^wt^: MPGKKARKNAQPSPARAPAELEVEC ATQLRRFGDKLNFRQKLLNLISKLFCSGT). Ubiquitin assays were performed in 1× E3 ubiquitin ligase buffer (Enzo) and 1× activation buffer (Enzo) for 1.5 h at 37 °C, unless indicated otherwise. Binding assays were conducted in PBS for 30 min at RT. In general, ubiquitylation assays were performed with 2 µg ubiquitin, 0.5 µg E2 (UbcH5a/b), 0.3 µg E1 (UBE1), 0.5 µg substrate (NOXA), and 0.4 µg E3 (CHIP) in a total amount of 20 µl reactions.

Subsequent experiments were conducted either with the above-mentioned recombinant proteins or with purified recombinant proteins generated using aLICator LIC Cloning and Expression System (ThermoScientific). In this case, NOXA::6×His and CHIP::6xHis were cloned into pLATE31 expression vector and transformed into *E. coli* BL21 cells. Expression of NOXA and CHIP were performed according to aLICator LIC Cloning and Expression System manufacturer’s instructions. Purification of polyhistidine-tagged NOXA and CHIP was performed using Protino Ni-TED columns (Macherey-Nagel) according to manufacturer’s instructions. Collectively, those ubiquitylation assays contained 60 µM ubiquitin or different ubiquitin variants (Boston Biochem) as indicated in figure legends, 100 nM E1 (UBA1), 1 µM E2 (UBE2H, UBE2R1, UBED1, UBE2D2, UBE2D3, UBE2E1, UBE2E3, UBE2L3, UBE2C, UBE2N-UBE2V1; Boston Biochem), 1 µM E3 (CHIP-6xHis, flag-XIAP (BPS Bioscience) or UFD-2-6xHis), and 1 µM of substrate proteins flag-NOXA (Origene), or NOXA-6×His mutants (wt, K4, 5, 8R, K35, 41, 48R, K0) or 1×His-MCL-1 (Abcam), unless otherwise indicated in figure legends. These ubiquitylation assays were performed in E3 Ligase Reaction Buffer (Boston Biochem) at 30 °C for 1 h and reactions were started by adding 1× Energy Regenerating Solution (Boston Biochem).

### Liquid chromatography-tandem mass spectrometry

For sample preparation, eluted NOXA-IP samples were incubated with 5 mM DTT for 1 h at 37 °C followed by incubation with 40 mM chloroacetamide (CAA) for 30 min at RT in a dark chamber. Samples were digested for 4 h at 37 °C with Lys-C. Prior to the addition of trypsin overnight at 37 °C, the urea concentration was diluted with 50 mM Tris-HCl (pH 8) to a final concentration of 2 M. Samples were acidified with acetic acid to a final concentration of 1% and purified using Styrene Divinyl Benzene (SDB)-StageTips.

For mass spectrometry, all samples were analyzed on a Q Exactive Plus (ThermoScientific) mass spectrometer that was coupled to an EASY nLC 1000 (ThermoScientific). Peptides were loaded with solvent A (0.1% formic acid in water) onto an in-house packed analytical column (50 cm–75 µm I.D., filled with 2.7 µm Poroshell EC120 C18, Agilent). Peptides were chromatographically separated at a constant flow rate of 250 nl/min using the following gradient: 3–5% solvent B (0.1% formic acid in 80% acetonitrile) within 1.0 min, 5–30% solvent B within 40.0 min, 30–50% solvent B within 8.0 min, 50–95% solvent B within 1.0 min, followed by washing and column equilibration. The mass spectrometer was operated in data-dependent acquisition mode. The MS1 survey scan was acquired from 300–1750 *m*/*z* at a resolution of 70,000. The top 10 most abundant peptides were isolated within a 1.8 Th window and subjected to HCD fragmentation at a normalized collision energy of 27%. The AGC target was set to 5e5 charges, allowing a maximum injection time of 110 ms. Product ions were detected in the Orbitrap at a resolution of 35,000. Precursors were dynamically excluded for 10.0 s.

For data analysis, all mass spectrometric raw data were processed with Maxquant (version 1.5.3.8) using default parameters. Briefly, MS2 spectra were searched against the UniProt HUMAN.fasta database, including a list of common contaminants. False discovery rates on protein and PSM levels were estimated by the target-decoy approach to 1% (Protein FDR) and 1% (PSM FDR), respectively. The minimal peptide length was set to 7 amino acids and carbamidomethylation at cysteine residues was considered as a fixed modification. Oxidation (M) and Acetyl (Protein N-term) were included as variable modifications. The match-between runs option was disabled. LFQ quantification was enabled using default settings. Further analysis was performed using Perseus software (v 1.5.5.3). Briefly, proteins flagged as “potential contaminants”, “reverse” or “identified by site” were excluded from the data set. LFQ values were log2 transformed and significant binders were defined as proteins not present in the IgG control.

### Cell death analysis

Cells were seeded at 20,000 cells per well on a 96-well plate and transfected with indicated siRNAs for 48 h. Cells were treated as indicated for 24 h in the presence of 5 μM SYTOX Green (ThermoFisher) or 5 μM CellEvent Caspase-3/7 Green Detection Reagent (Invitrogen). Dead cells were imaged in real-time for the indicated duration of time in either 1 h intervals (SYTOX Green) or 20 min intervals (CellEvent Caspase-3/7) via fluorescence signals using an IncuCyte S3 Live-Cell Analysis System (Essen Bioscience). Resulting images were analyzed using the software package (IncuCyte 2019B Rev) of the IncuCyte, which allows quantification of positive cells normalized to all cells per well following the basic analysis program. For time-lapse experiments, the average of each well is applied, whereas for cell death analysis after 24 h, single values of each well are presented. Data are presented as fold change in relation to untreated control.

### Statistical analysis

qPCR values are presented as mean ± standard error of the mean (SEM). Samples sizes were chosen according to the basis of previous publications without prior power analysis. Significance was determined using one-way ANOVA with Bonferroni’s multiple comparisons test. Significances were indicated as ^ns^*p* > 0.05, *p ≤ 0.05, **p ≤ 0.01, ***p ≤ 0.001.

## Results

### DNA damage-induced NOXA expression is accompanied by NOXA ubiquitylation involving CHIP

**Fig. 1:**
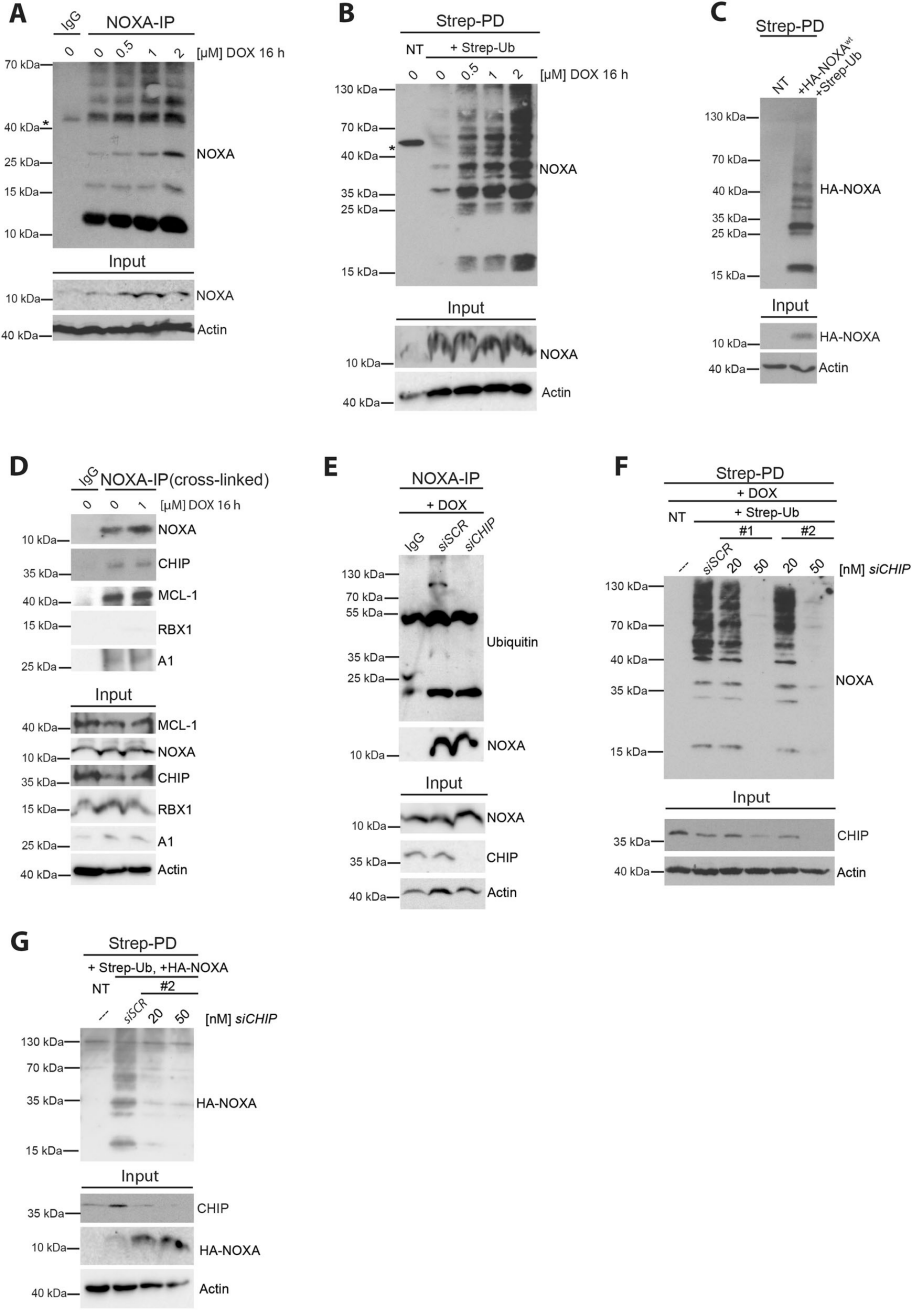
NOXA is ubiquitylated upon DNA damage. Western blot analysis of cell lysate (Input) and **a** NOXA-IP of HeLa cells treated with indicated DOX concentrations for 16 h, **b** Strep-PD of HEK293T cells transfected with Strep-Ub^wt^ for 36 h and treated with indicated DOX concentrations for 16 h, **c** Strep-PD of HeLa cells transfected with Strep-Ub^wt^ and HA-NOXA^wt^, or **d** NOXA-IP of HeLa cells treated with DOX as indicated. Cells were crosslinked via disuccinimidyl suberate (DSS) prior to lysis. **e** NOXA-IP of HEK293T cells transfected with *siSCR* or *siCHIP* (#2) as indicated for 48 h. All samples were treated with 2 µM DOX for 16 h prior to lysis. **f** Strep-PD of HEK293T cells transfected with Strep-Ub^wt^ and *siSCR* or siRNAs #1 and #2 targeting *CHIP* as indicated for 48 h. All samples were treated with 1 µM DOX for 16 h prior to lysis. **g** Strep-PD of HeLa cells transfected with Strep-Ub^wt^, HA-NOXA^wt^, and siRNA#2 targeting *CHIP* for 48 h. Asterisk (*) indicates a non-specific band. Actin served as a loading control in all experiments. NT not transfected. DOX doxorubicin. All experiments are representatives of at least three independent experiments.

DNA damage is considered as one of the main drivers of NOXA expression^1,29^. Our data show that both NOXA transcript and protein levels are elevated in response to genotoxic stress induced by the anthracycline drug doxorubicin (DOX) in a dose- and time-dependent manner (Fig. 1a and Supplementary Fig. S1A). The experimental setup (dosage and exposure time) used in these analyses did not induce cellular death and was not altered by caspase inhibition (Supplementary Fig. S1B). In addition to the calculated molecular mass of NOXA protein (~10 kDa), immunoprecipitation with NOXA-specific antibody revealed an accumulation of higher molecular weight forms of NOXA, suggesting that NOXA protein is modified in response to DNA damage (Fig. 1a). To assess NOXA ubiquitylation, cells ectopically expressing Strep-tagged Ub (Strep-Ub) were exposed to DOX and ubiquitylated proteins were immobilized by Strep-Tactin following Strep-Ub pulldown (PD). Indeed, endogenous NOXA was increasingly ubiquitylated in response to genotoxic stress in a dose- and time-dependent manner (Fig. 1b and Supplementary Fig. S1C). Similar results were obtained when cells were exposed to genotoxic stress induced by the topoisomerase II inhibitor etoposide (ETO) (Supplementary Fig. S1D), but not when treated with the protein kinase inhibitor staurosporine (STS), a well-known inducer of mitochondrial apoptotic cell death, which did not stimulate NOXA accumulation (Supplementary Fig. S1E). Like endogenous NOXA, ectopically overexpressed HA-tagged NOXA (HA-NOXA) was also ubiquitylated even in the absence of DNA damage (Fig. 1c). This indicates that NOXA protein biosynthesis is accompanied by ubiquitylation processes irrespective of the source of stimulus that drives NOXA expression.

To identify the E3 Ub ligase regulating NOXA ubiquitylation, we performed mass spectrometric (MS) analysis of immunoprecipitated endogenous NOXA as well as ectopically expressed HA-NOXA as bait under non-denaturating conditions. Besides NOXA, two E3 Ub ligases, namely CHIP^26^ and RBX1^30^, were identified in the MALDI-TOF MS analysis of NOXA precipitates (Supplementary Table 1). Immunoprecipitation (IP) assay using a NOXA-specific antibody to pulldown endogenous NOXA revealed co-immunoprecipitation of CHIP but not RBX1 (Fig. 1d). In addition, we detected co-precipitated MCL-1 and A1 as functional counterparts of NOXA. Corresponding to the co-immunoprecipitation data, siRNA-mediated knockdown of CHIP significantly reduced the ubiquitylation of endogenous NOXA in DOX-treated cells (Fig. 1e, f), whereas downregulation of RBX1 did not impact on NOXA ubiquitylation (Supplementary Fig. S1F). Ubiquitylation of ectopically expressed HA-NOXA was also efficiently reduced upon the siRNA-mediated knockdown of CHIP (Fig. 1g). Notably, we did not observe a marked upregulation of *CHIP* transcript (Supplementary Fig. S1G) or protein (Fig. 1d) in response to DNA damage. Taken together, our results provide strong evidence that CHIP is required for the ubiquitylation of NOXA upon genotoxic stress.

### The E3 ubiquitin ligase CHIP directly ubiquitylates NOXA

**Fig. 2:**
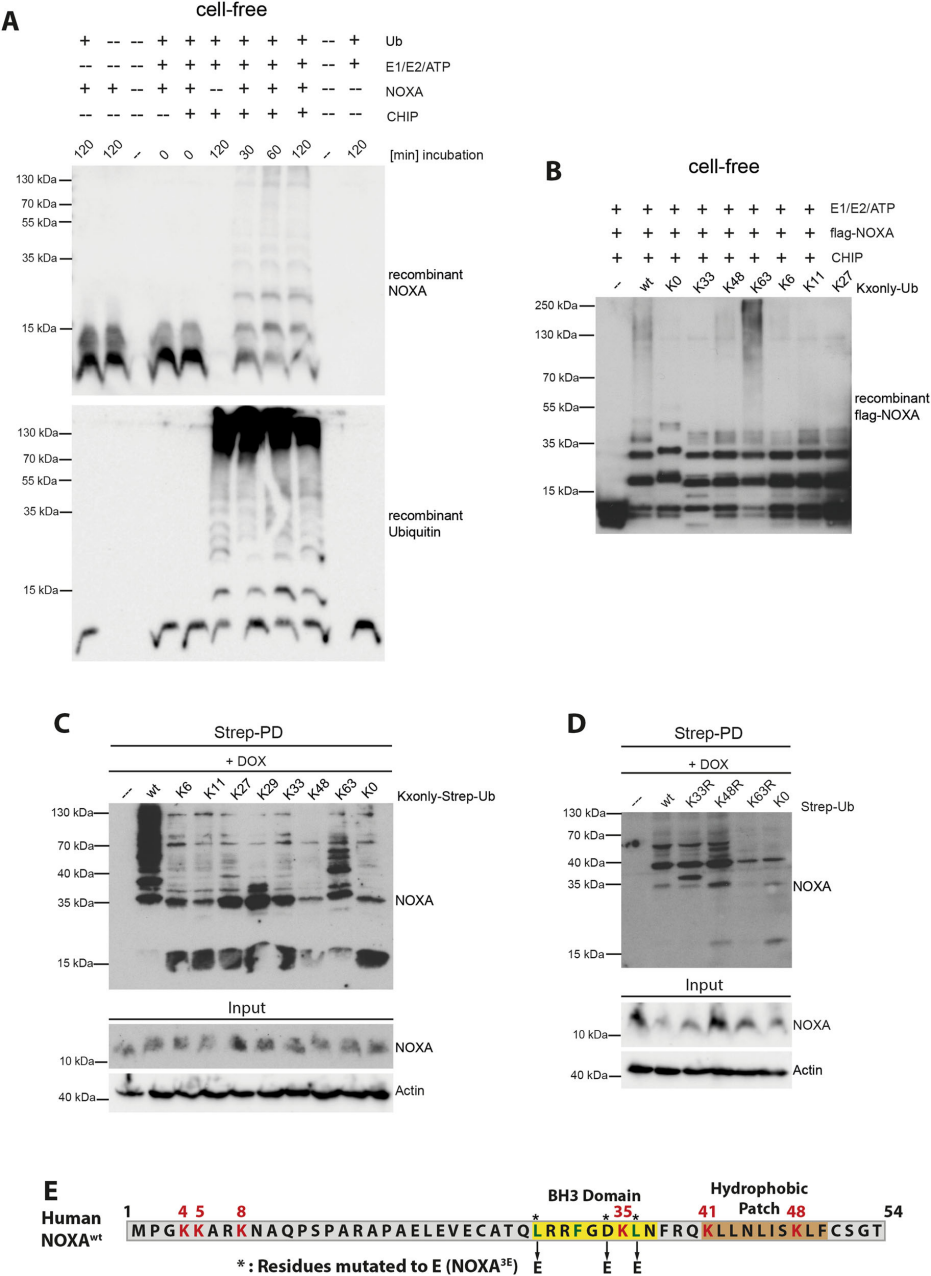
NOXA is conjugated with K63 Ub chains by CHIP. Cell-free ubiquitylation assay of **a** Recombinant NOXA incubated with recombinant CHIP for indicated time points. **b** Recombinant CHIP and flag-tagged NOXA incubated with Ub chain-assembly mutants (Kxonly) as indicated. Western blot analysis of cell lysate (Input) and **c** Strep-PD of HeLa cells transfected with Strep-Ub chain-assembly mutants (Kxonly) for 36 h and treated with 1 µM DOX for 16 h. **d** Strep-PD of HeLa cells transfected with Strep-Ub chain-assembly mutants (KxR) as indicated for 36 h and treated with 1 µM DOX for 16 h. **c**, **d** Actin served as a loading control. **e** Amino acid sequence of human NOXA harboring one BH3 domain (yellow) and a hydrophobic patch (brown) at the C terminus of the protein. Lysine residues (K) are marked and numbered in red, conserved residues of the BH3 domain are depicted in green and mutated residues are marked with asterisks (3E). NT not transfected. DOX doxorubicin. All experiments are representatives of at least three independent experiments.

To investigate whether CHIP can directly ubiquitylate NOXA, we employed a cell-free ubiquitylation assay using different recombinant E2 enzymes^24^. CHIP efficiently ubiquitylated recombinant NOXA or flag-tagged NOXA (flag-NOXA) in a time-dependent manner, specifically when co-incubated with the E2 enzymes UbcH5a, UbcH5b, or UbcH5c (Fig. 2a and Supplementary Fig. S2A). In contrast to CHIP, neither the UbcH5 family-compatible E3 Ub RING ligase XIAP^31^ nor the E3 Ub U-box ligase UFD-2^32^ was able to poly-ubiquitylate NOXA (Supplementary Fig. S2B). Our results revealed only mono- or di-ubiquitylation of NOXA by UFD-2 or XIAP. Together, these data identify CHIP as a bona fide E3 Ub ligase of NOXA.

Ubiquitylation occurs primarily on internal lysine residues and defines the fate of the conjugated substrate proteins based on the topology defined by the specific linkage type of the Ub conjugates^33^. Our cell-free ubiquitylation analysis with different recombinant Ub protein variants possessing only one lysine residue (Kxonly) revealed that recombinant CHIP preferentially ubiquitylated NOXA via K63-linked Ub (Fig. 2b). In addition, weak K48-linked Ub polymeric chains were detected, while Ub variants only possessing K6, K11, K27, K33, or lacking all lysine residues (K0) were not incorporated in poly-Ub chains attached to NOXA by CHIP. These data were further substantiated in cells ectopically expressing Kxonly Strep-Ub variants and exposed to genotoxic stress (Fig. 2c). In line with our cell-free analysis, endogenous NOXA was mainly but not exclusively modified by K63-linked ubiquitin chains. Moreover, K29-, K33-, and K48-linked Ub modifications of endogenous NOXA were only detectable upon treatment with the proteasome inhibitor Bortezomib (BORTE) (Supplementary Fig. S2C), which might also be advantaged by combined overexpression of ubiquitin mutants and proteasome inhibition. Complementary analysis by ectopic expression of Strep-Ub variants lacking either K33 (K33R), K48 (K48R), K63 (K63R), or all lysine residues (K0) revealed that only mutation of the K63 lysine residue within Ub caused a significant reduction in endogenous NOXA ubiquitylation in cells exposed to DOX (Fig. 2d).

NOXA comprises six lysine residues (K4, K5, K8, K35, K41, and K48) that can potentially serve as sites of Ub conjugation (Fig. 2e). To identify the lysine residue(s) of NOXA utilized by CHIP for ubiquitylation, we ectopically expressed HA-NOXA mutants in which individual lysine residues were mutated to arginine (R) either inclusively (K0), only at one position (lacking one lysine residue, KxR), or with the exception of one position (possessing only one single lysine residue, Kxonly) and examined HA-NOXA ubiquitylation by Strep-Ub PD. In line with a previous study^10^, this analysis identified three specific lysine residues K35, K41, and K48 that are ubiquitylated (Supplementary Fig. S2D, E). Only a combined mutation of all three identified lysine residues fully diminished NOXA ubiquitylation in cells (Supplementary Fig. S2F). Similarly, in a cell-free ubiquitylation assay using recombinant CHIP our data revealed that whereas simultaneous mutation of K4, K5, and K8 did not impact on recombinant NOXA ubiquitylation, NOXA ubiquitylation was completely abolished when lysine residues K35, K41, and K48 of NOXA were mutated to arginine (R) (Supplementary Fig. S2G). Together, these findings demonstrate that genotoxic stress-induced NOXA ubiquitylation is preferentially mediated by CHIP via K63-linked Ub chains by utilizing K35, K41, and K48 lysine residues of NOXA.

### K63-linked ubiquitylation tags NOXA for lysosomal degradation

**Fig. 3:**
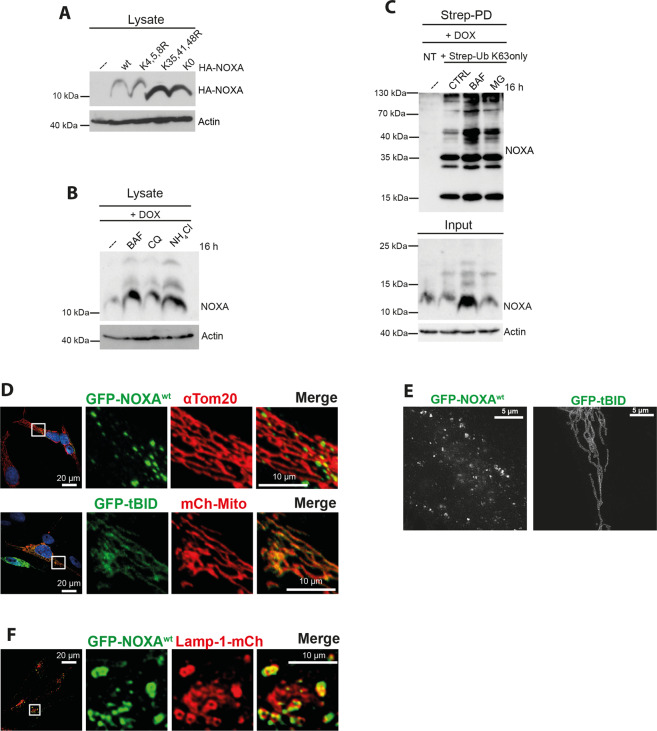
NOXA gets degraded at lysosomes. Western blot analysis of HeLa cell lysates **a** transfected with HA-NOXA lysine mutants (KxxxR) as indicated for 36 h, **b** treated with 100 nM BAF, 10 µM CQ and 30 mM NH_4_Cl for 16 h, as indicated. **c** Western blot analysis of cell lysate (Input) and Strep-PD of HEK293T cells transfected with Strep-Ub K63 only (CTRL) for 36 h and treated with 100 nM BAF or 5 µM MG132 for 16 h. **a**–**c** Actin served as a loading control. **d** Confocal microscopy of HeLa cells transfected with GFP-NOXA^wt^ or GFP-tBID for 16 h and mitochondria were stained with αTom20 antibody (568 nm) or co-transfected with mCh-Mito construct. Nuclei were counterstained with DAPI. **e** STED microscopy of HeLa cells transfected with GFP-NOXA^wt^ or GFP-tBID for 16 h. **f** Confocal microscopy of HeLa cells transfected with GFP-NOXA^wt^ and Lamp-1-mCh for 16 h. NT not transfected. DOX doxorubicin. CQ chloroquine. BAF bafilomycin. MG MG132. mCh mCherry. Mito Mitochondria. All experiments are representatives of at least three independent experiments.

Notably, our data indicate that NOXA was preferentially ubiquitylated via K63-linked Ub chains (Fig. 2), which usually do not promote proteasomal degradation^34^. Although K63-linked Ub chains have been mainly described to be involved in non-degradative processes^35^, they have also been involved in protein degradation by endo-lysosomes^36^. In particular, CHIP has been previously shown to induce Ub-dependent lysosomal degradation of cytosolic, vesicular or transmembrane proteins upon their modification with different Ub linkages, including K63-linked Ub chains^37–40^. Indeed, both HA-NOXA^K35,41,48R^ and HA-NOXA^K0^ lacking lysine residues required for ubiquitylation, exhibited significantly higher protein levels compared to HA-NOXA (Fig. 3a and Supplementary Fig. S2F). Furthermore, treatment with either bafilomycin A1 (BAF), an inhibitor of lysosomal acidification/maturation, or the lysosomotropic reagents chloroquine (CQ) and NH_4_Cl, resulted in the accumulation of endogenous NOXA upon DOX treatment (Fig. 3b). Additional analysis of cells ectopically expressing K63-only Strep-Ub showed that treatment with BAF stabilized and increased the abundance of K63-linked ubiquitylated endogenous NOXA upon DNA damage. In contrast, the proteasome inhibitor MG132 had no obvious impact (Fig. 3c). Together, these data indicate that the K63-linked ubiquitylation of NOXA by CHIP initiates its lysosomal degradation.

Our initial immunoprecipitation studies combined with MALDI-TOF MS analysis of NOXA precipitates already suggested the association of NOXA with a number of endo-lysosomal factors (Supplementary Table 1). To examine the association of NOXA with the lysosomal compartment we carried out a detailed analysis of subcellular localization of NOXA using confocal, stimulated emission depletion (STED), and electron microscopy (EM). Our confocal microscopical analysis revealed a dot-like cytoplasmic distribution of overexpressed untagged NOXA or GFP-tagged NOXA (Supplementary Fig. S3A and Fig. 3d), which was further confirmed by STED microscopy (Fig. 3e). The subcellular distribution pattern of NOXA differed distinguishably from mitochondrial localization of GFP-tagged truncated BID (GFP-tBID), another pro-apoptotic member of the BH3-only protein family (Fig. 3d, e). Furthermore, our EM analysis of cells expressing NOXA, which was N-terminally fused to an ascorbate peroxidase 2 (APEX2) tag (APEX2-NOXA) providing enhanced EM contrast^41^, showed a vesicular association of NOXA (Supplementary Fig. S3B). Subsequently, co-localization studies with a set of specific markers of vesicular compartments suggested that NOXA (tagged and untagged) co-localizes with late endosomal and lysosomal markers such as Rab9, Rab7, and Lamp-1 (Supplementary Fig. S3C, D and Fig. 3f). Despite the distinct co-localization and identification as co-precipitates with NOXA in our MS analysis (Supplementary Tab. 1), our additional immunoprecipitation studies did not support a direct interaction of NOXA with endo-lysosomal markers (Supplementary Fig. S3E). This discrepancy is due to the fact that in our MS analysis cells were crosslinked for optimizing MS outcome. This may cause co-precipitation of proteins with close proximity but not necessarily direct interaction (e.g., co-localization). Furthermore, although displaying broad cytosolic distribution, untagged NOXA^K0^ and mCherry-NOXA^K0^ did neither assemble with immunostained Lamp-1 nor with GFP-Rab7 or mitochondrial markers indicating that ubiquitylation triggers the lysosomal targeting of NOXA but is not involved in the failure of NOXA to associate with mitochondria (Supplementary Fig. S3F). Together, these data support the notion that the ubiquitylation of NOXA by CHIP initiates its lysosomal degradation.

### MCL-1 counteracts NOXA ubiquitylation by CHIP and directs mitochondrial localization of NOXA

**Fig. 4:**
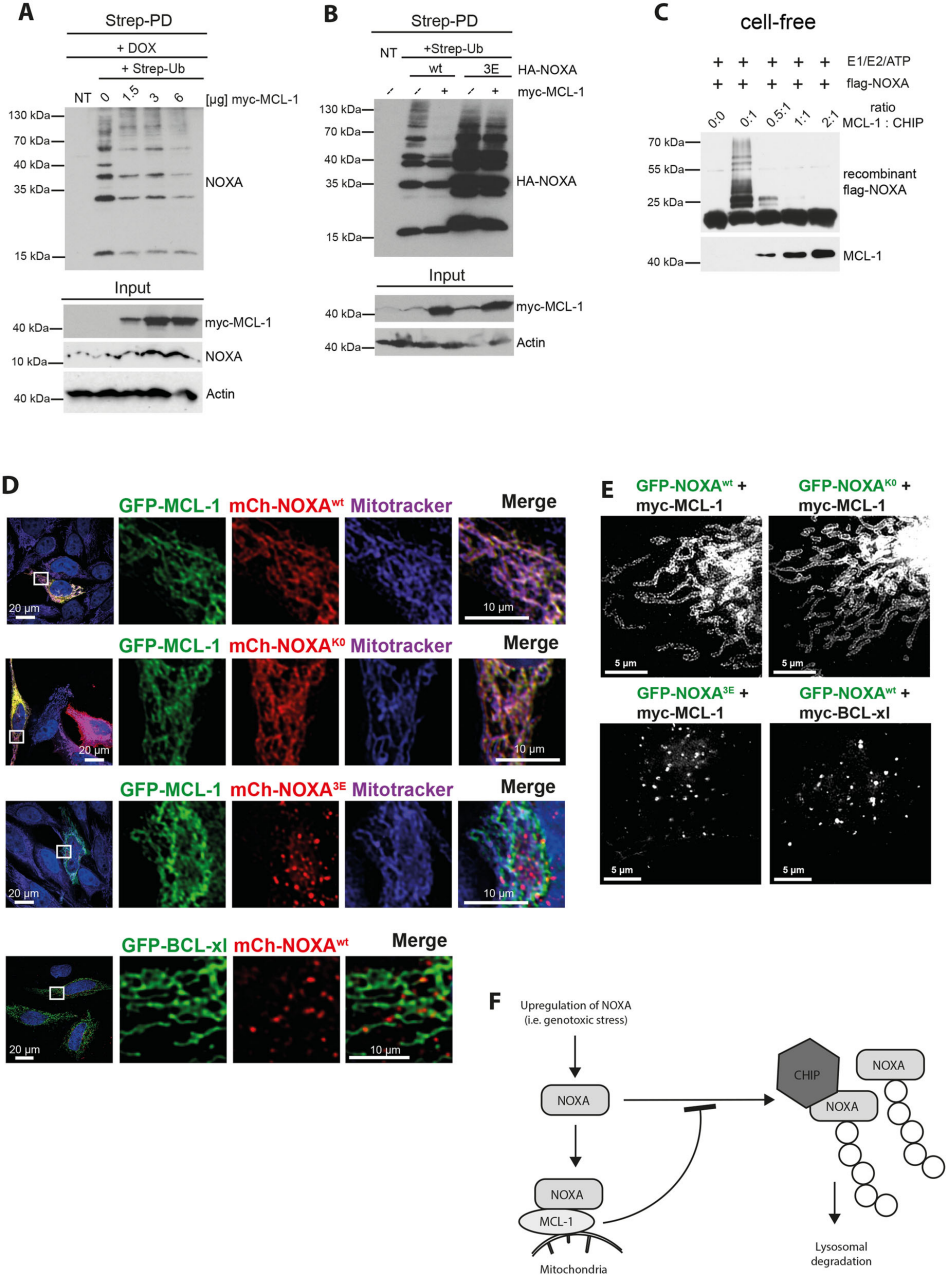
MCL-1 binding prevents NOXA ubiquitylation. Western blot analysis of cell lysate (Input) and **a** Strep-PD of HeLa cells transfected with Strep-Ub^wt^ and increasing amounts of myc-MCL-1 as indicated for 36 h and treated with 1 µM DOX for 16 h, **b** Strep-PD of HeLa cells transfected with Strep-Ub^wt^, HA-NOXA^wt^, HA-NOXA^3E^, and myc-MCL-1 for 36 h as indicated. **c** Cell-free ubiquitylation assay of recombinant protein incubating flag-tagged NOXA, CHIP, and MCL-1 as indicated. **d** Confocal microscopy of HeLa cells transfected with mCh-NOXA^wt^, mCh-NOXA^K0^, or mCh-NOXA^3E^, and GFP-MCL-1 or GFP-BCL-xl as indicated for 16 h and mitochondria were visualized by mitotracker (633 nm). Nuclei were counterstained with DAPI. **e** STED microscopy of HeLa cells transfected with GFP-NOXA^wt^, GFP-NOXA^K0^, or GFP-NOXA^3E^, and myc-MCL-1 or myc-BCL-xl as indicated for 16 h. **f** Schematic diagram of CHIP ubiquitylating abundant NOXA. Upon DNA damage, NOXA is transcriptionally upregulated and targeted to mitochondria through MCL-1 binding. Accelerated levels of NOXA beyond the protein amount of MCL-1 are targeted for lysosomal degradation by CHIP-mediated ubiquitylation utilizing K63-linked Ub chains. NT not transfected. DOX doxorubicin. mCh mCherry. All experiments are representatives of at least three independent experiments.

Our results provide compelling evidence that CHIP directly binds and ubiquitylates NOXA at lysine residues located in its BH3 domain (K35) and the hydrophobic patch (K41, K48) (Figs. 1 and 2). Notably, the interaction of NOXA and MCL-1 requires an unmasked BH3 domain and thus, NOXA ubiquitylation may interfere with its capability to bind MCL-1. Our data using ectopically expressed myc-tagged MCL-1 (myc-MCL-1) showed that MCL-1 efficiently blocked NOXA ubiquitylation and stabilized endogenous NOXA as well as HA-NOXA (Fig. 4a and Supplementary Fig. S4A). In contrast to MCL-1, co-expression of the anti-apoptotic protein myc-BCL-xl, which does not bind to NOXA^3^ (Supplementary Fig. S4B), did not reduce HA-NOXA ubiquitylation (Supplementary Fig. S4C). Furthermore, HA-NOXA^3E^ (Fig. 2e), which is unable to bind MCL-1^3^, was highly ubiquitylated even after myc-MCL-1 overexpression (Fig. 4b and Supplementary Fig. S4D), indicating that MCL-1 binding blocks NOXA ubiquitylation. To test this hypothesis, we performed a cell-free ubiquitylation assay using recombinant CHIP, NOXA, and MCL-1 protein. The pre-incubation of NOXA with increasing concentrations of MCL-1 efficiently reduced the ubiquitylation of NOXA by CHIP (Fig. 4c). Of note, MCL-1 itself was not a target for CHIP-mediated ubiquitylation (Supplementary Fig. S4E). These data collectively indicate that NOXA is primarily dedicated to binding to MCL-1 as its presence efficiently blocked NOXA ubiquitylation and degradation. In line with this notion, the specific knockdown of MCL-1 in cells increased the ubiquitylation of HA-NOXA and decreased its stability (Supplementary Fig. S4F).

We further examined whether MCL-1 also impacts on the subcellular localization of NOXA. Confocal and STED microscopical analyses revealed that the presence of ectopically expressed GFP-MCL-1 provoked mitochondrial localization of NOXA (mCherry-tagged and untagged) and NOXA^K0^ (Fig. 4d, e and Supplementary Fig. S4G) whereas no NOXA co-localization was observed with the lysosomal marker Lamp-1 (Supplementary Fig. S4H). Mitochondrial localization of NOXA upon co-expression with MCL-1 was additionally confirmed using EM with the expression of APEX2-NOXA together with myc-MCL-1 (Supplementary Fig. S4I). The mitochondrial distribution of NOXA was exclusively dependent on the interaction with MCL-1 since the mCherry-tagged BH3 NOXA mutant (mCherry-NOXA^3E^) defective in MCL-1 binding did not show any mitochondrial localization upon GFP-MCL-1 co-expression (Fig. 4d, e). Similarly, overexpression of GFP-BCL-xl also failed to promote mitochondrial localization of mCherry-NOXA (Fig. 4d, e), providing additional evidence that mitochondrial localization of NOXA requires its binding to MCL-1. Of note, both MCL-1 and BCL-xl overexpression revealed mitochondrial distribution as shown by co-localization with Tom20 as a mitochondrial marker or in STED microscopy (Supplementary Fig. S4J, K).

Our findings explored a regulatory mechanism that controls the fate of cytosolic NOXA during cellular stress responses such as genotoxic stress (Fig. 4f). We showed that CHIP ligase activity triggers the lysosomal degradation of NOXA protein exceeding a certain threshold, which is defined by the expression level of MCL-1. MCL-1 binds and sequesters cytosolic NOXA to mitochondria and thereby efficiently blocks its accessibility for CHIP. CHIP, in turn, mediates the ubiquitylation of NOXA when it can not be engaged in the apoptotic process. Accordingly, a specific knockdown of CHIP in cells only slightly increased apoptosis, which was restored after an additional knockdown of NOXA (Supplementary Fig. S5A). This is in line with the fact that both overexpressed NOXA and NOXA^K0^ did not co-localize with mitochondria (Fig. 3d and Supplementary Fig. S3F) and were not capable to induce apoptotic cell death (Supplementary Fig. S5B). These data collectively suggest that the balanced control of NOXA serves as a cellular safeguard system to prevent cytosolic protein accumulation during cellular stress responses.

## Discussion

BH3-only proteins represent a distinct and structurally diverse class of proteins that serve as death sentinels and transmit apoptotic signals to the core BCL-2 family proteins^42,43^. Some BH3-only proteins are constitutively expressed whereas others including NOXA are expressed in response to stress cues, such as DNA damage. A large body of evidence shows that constitutively expressed BH3-only proteins remain inactive until an apoptotic trigger initiates their function through post-translational mechanisms such as proteolytic processing, e.g., required for the activation of BID yielding its active form tBID^12,44^. NOXA has been extensively studied in cancer and proteasome inhibitors have been shown to rely on NOXA for their therapeutic potential indicating that NOXA is a substrate of the ubiquitin–proteasome system (UPS)^2,9,10,14,45^. Of note, besides the reported cancer-associated alterations of the UPS that involve the Ub conjugation machinery, one previous study showed that overexpressed NOXA can act as a sensor for proteasome integrity and is degraded by the UPS by an Ub-independent process, which can be blocked by MCL-1^46^. Whereas the majority of previous results deal with the turn-over of NOXA and the alteration of UPS in cancer, recent studies explored a novel view about the fate of NOXA/MCL-1 protein complex on mitochondria. These data showed that the turn-over of NOXA/MCL-1 protein complex is efficiently regulated by mitochondrial quality control and dynamics machinery involving MARCH5^17–19^. Our data identified another mode in controlling the cytosolic pool of NOXA, which is not bound by MCL-1.

CHIP represents a central regulator of proteostasis that governs the biogenesis, folding, localization, and turn-over of various cellular proteins^47^. Our results conclusively demonstrate that NOXA is a physiologic target of CHIP, which efficiently controls NOXA level in response to DNA damage by K63-linked poly-ubiquitylation, which directs its lysosomal degradation. The association or proximity of NOXA to endo-lysosmal compartment/markers was first identified in our MS studies. Further extensive subcellular co-localization studies together with EM studies conclusively confirmed the lysosomal targeting of NOXA. Consistently, a recent study described that CHIP ubiquitylates and directs lysosomal degradation of the cytosolic receptor-interacting protein kinase 3 (RIPK3), an effector of necroptosis^38^. Together, these observations highlight the role of CHIP and lysosomal degradation machinery as a post-translational regulator of factors involved in different cell death pathways.

Given that NOXA specifically binds only to a subset of pro-survival BCL-2 proteins such as MCL-1 and A1 with high affinity, this selective binding only promotes cytotoxicity when co-expressed with its complementary BCL-2 member. Moreover, the tolerable expression level of a selective pro-apoptotic BH3-only protein seems to be defined by its specific anti-apoptotic binding partner, and protein levels that exceed this threshold should be removed to avoid protein accumulation. Given the fact that MCL-1 binds and inhibits NOXA ubiquitylation, our data discovered CHIP as a central regulator of the free pool of NOXA not bound by MCL-1 and thus not yet engaged in the apoptotic program.

## Supplementary information

Supplementary Figure S1

Supplementary Figure S2

Supplementary Figure S3-1

Supplementary Figure S3-2

Supplementary Figure S4-1

Supplementary Figure S4-2

Supplementary Figure S5

Supplementary Figure Legends

Supplementary Table 1

## Data Availability

The authors declare that the data supporting the findings of this study are available within the paper and its supplementary information files.
