# Supplementary figures and images for: CHIP ubiquitylates NOXA and induces its lysosomal degradation in response to DNA damage
Source: Cell Death Dis. 2020 Sep 10;11(9):740. doi: 10.1038/s41419-020-02923-x (PMC7484759; doi:10.1038/s41419-020-02923-x)

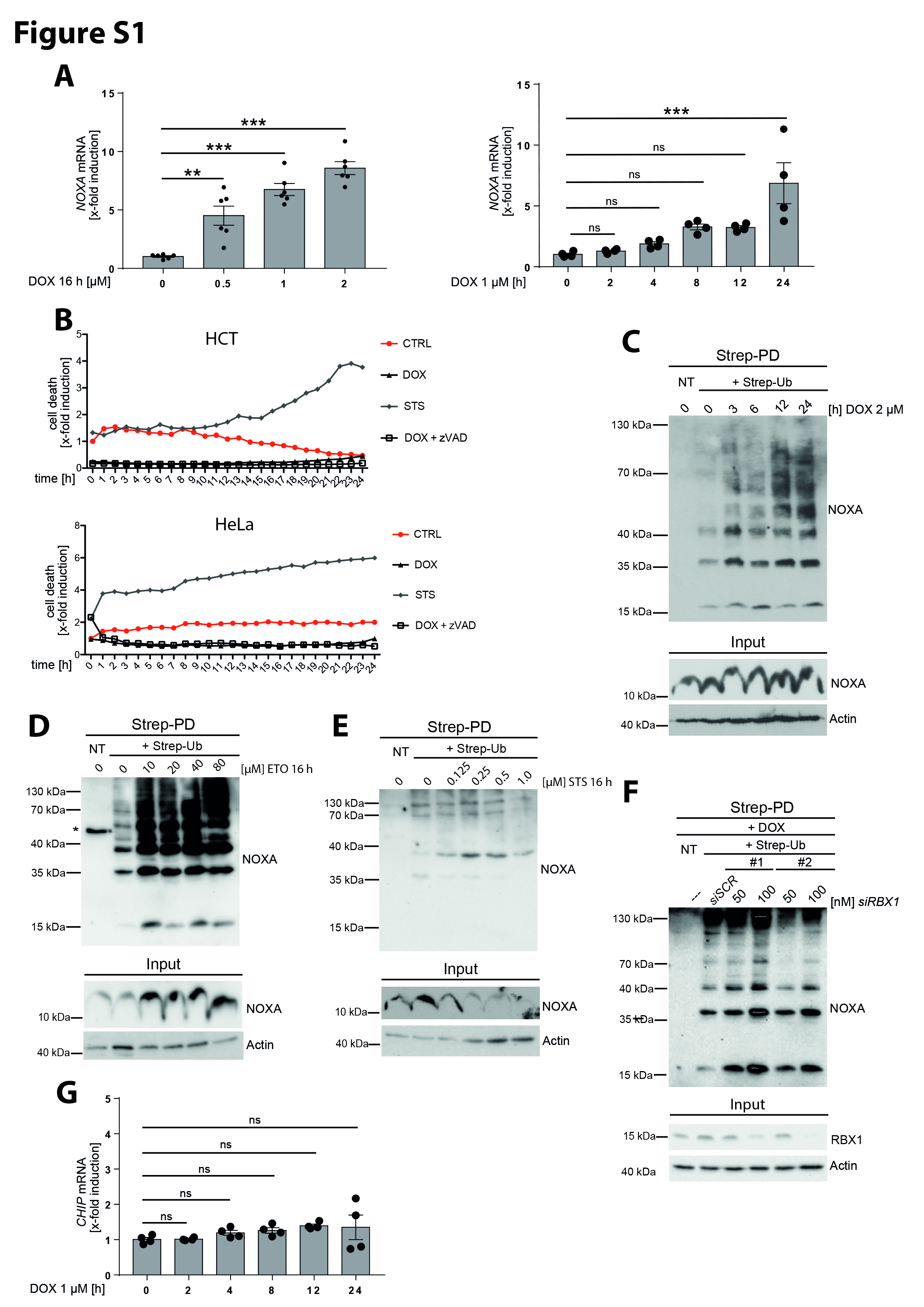

Supplement: Supplementary file 1 — Supplementary Figure S1 [file 41419_2020_2923_MOESM1_ESM.tif]

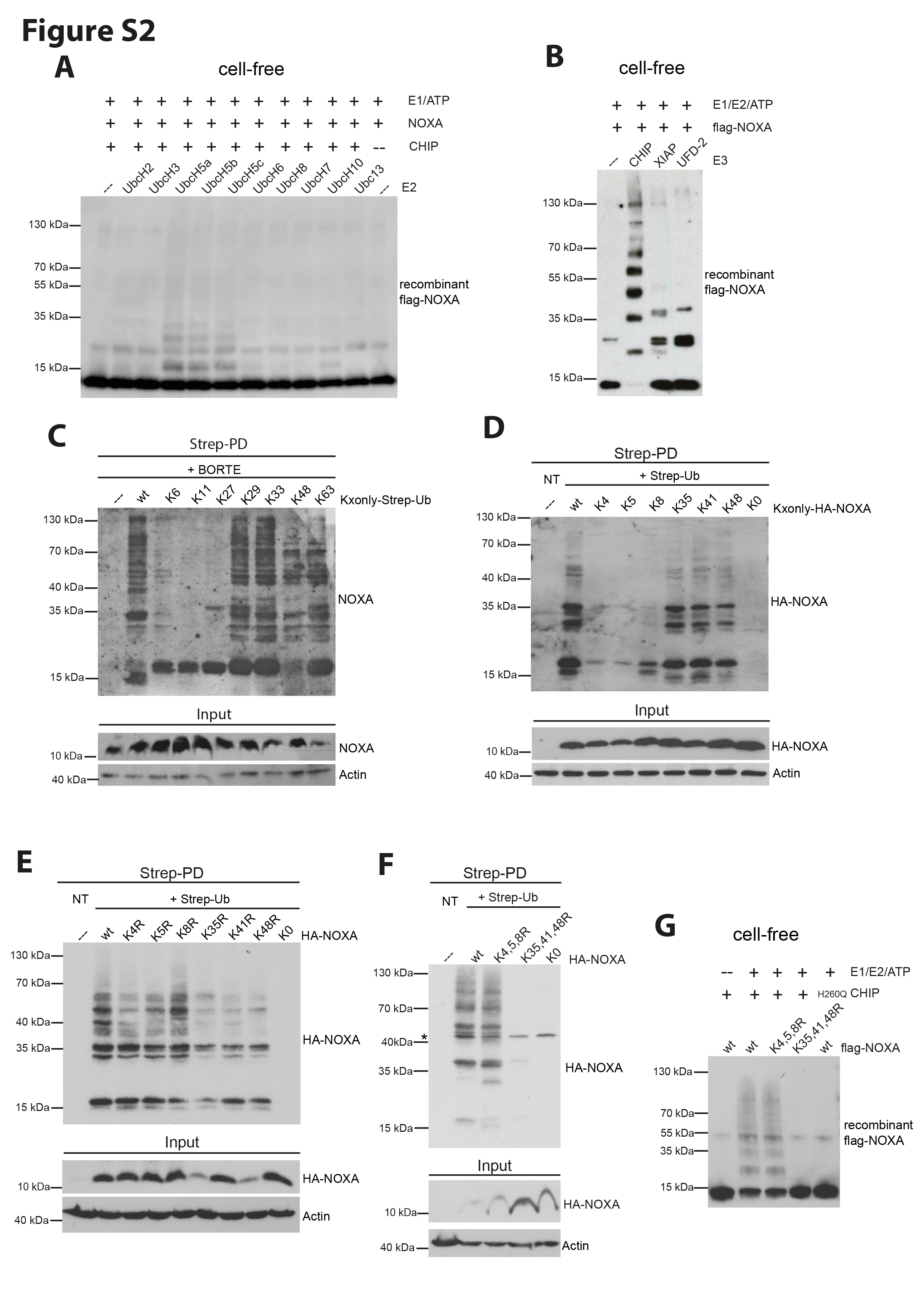

Supplement: Supplementary file 2 — Supplementary Figure S2 [file 41419_2020_2923_MOESM2_ESM.tif]

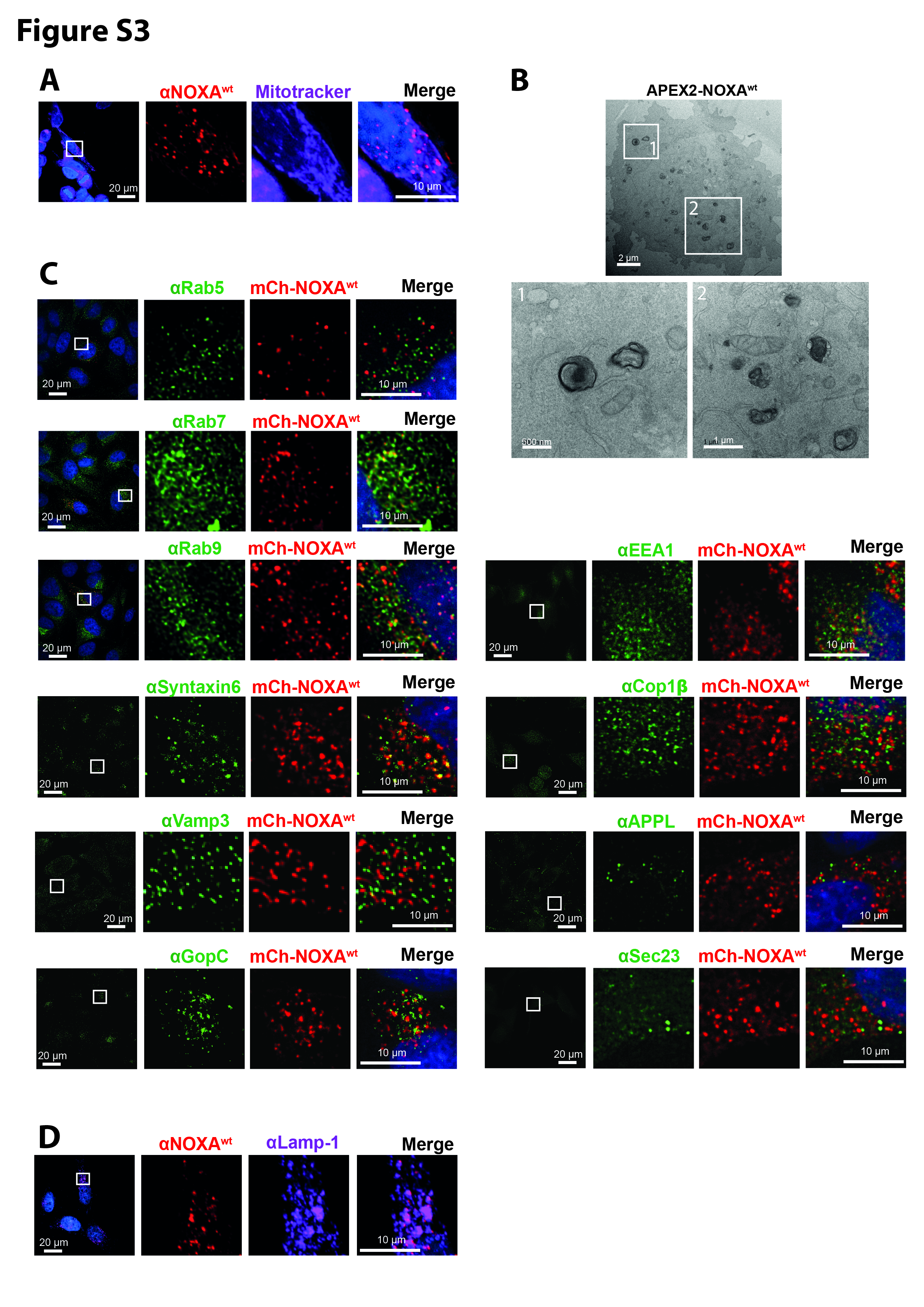

Supplement: Supplementary file 3 — Supplementary Figure S3-1 [file 41419_2020_2923_MOESM3_ESM.tif]

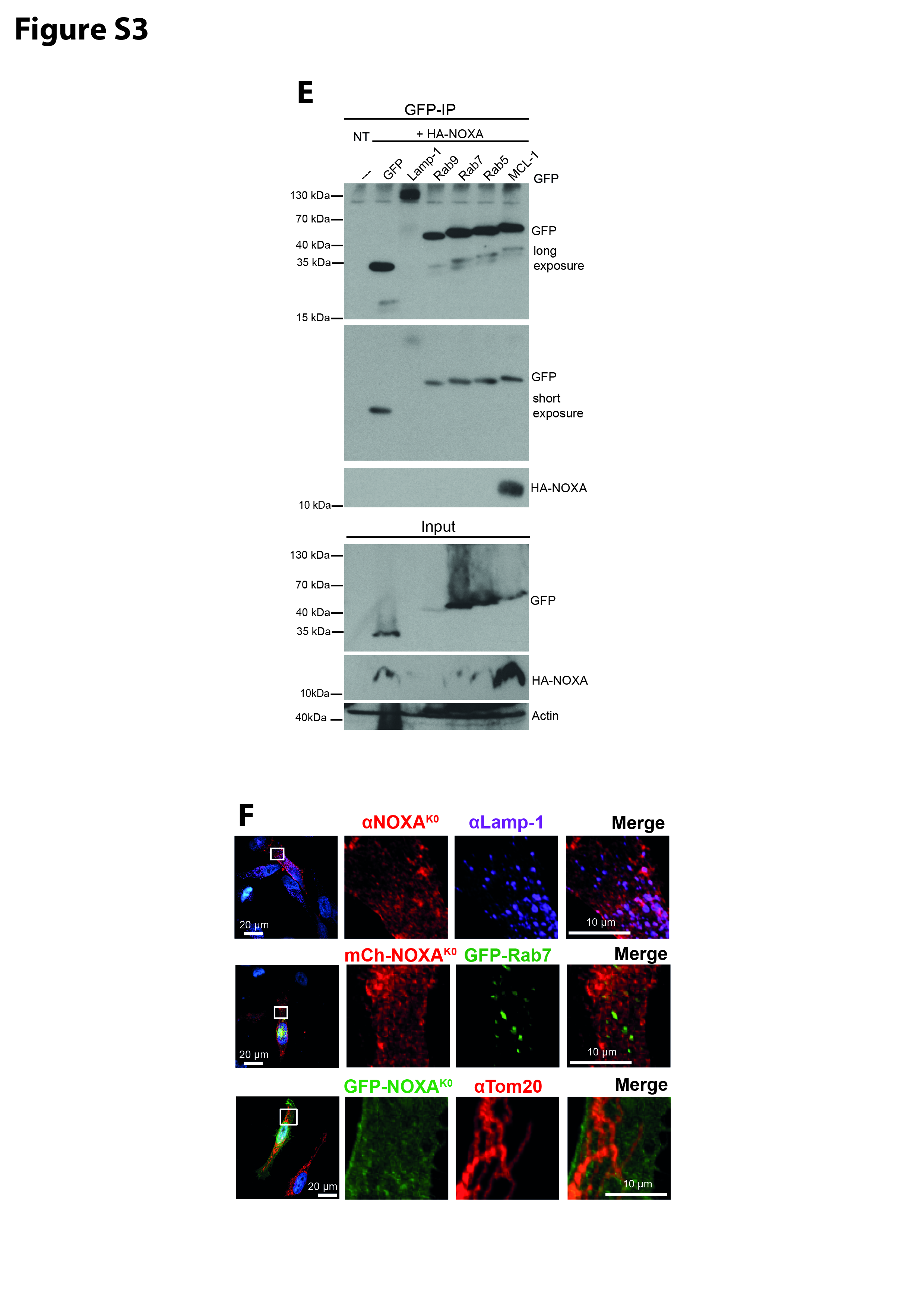

Supplement: Supplementary file 4 — Supplementary Figure S3-2 [file 41419_2020_2923_MOESM4_ESM.tif]

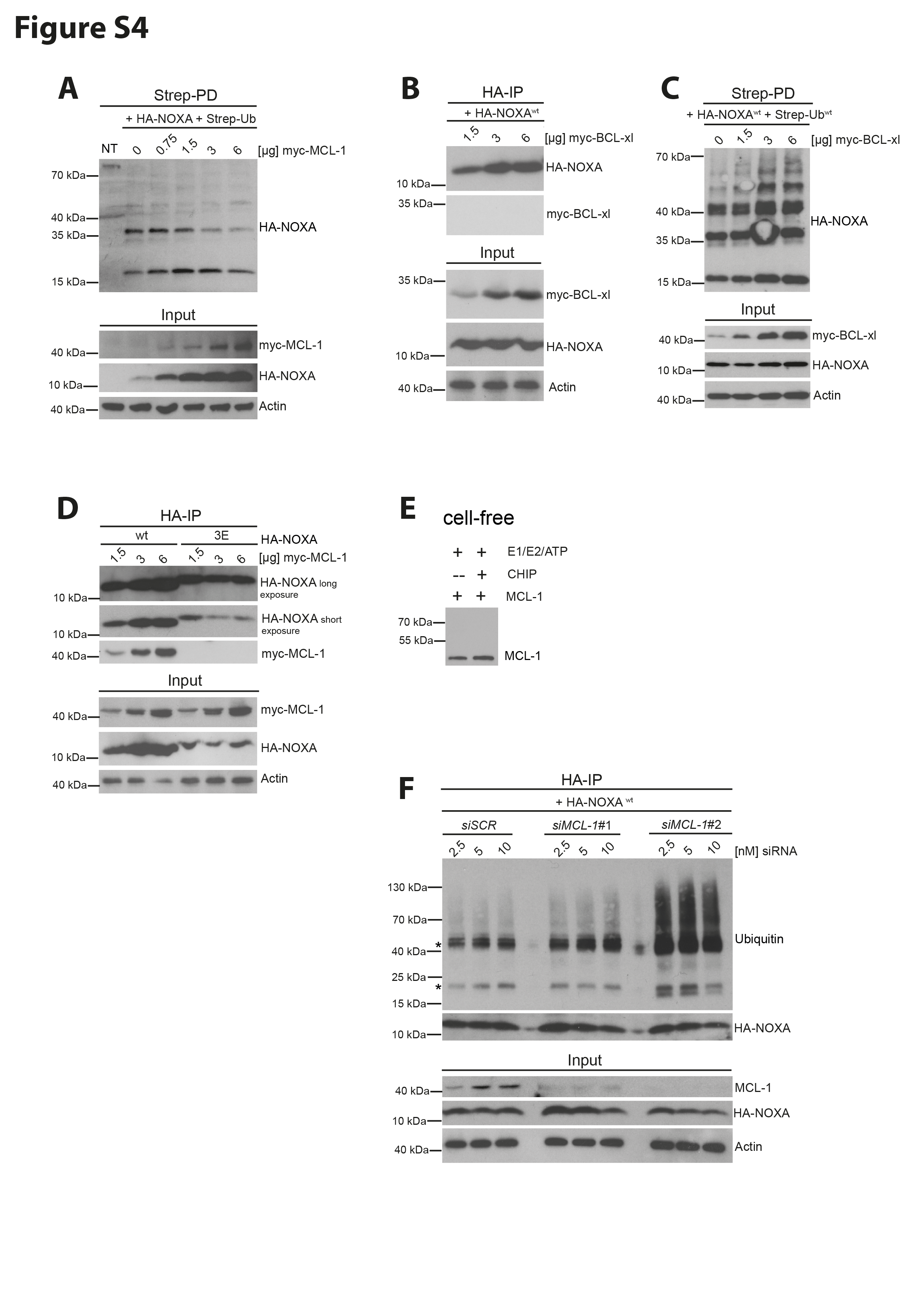

Supplement: Supplementary file 5 — Supplementary Figure S4-1 [file 41419_2020_2923_MOESM5_ESM.tif]

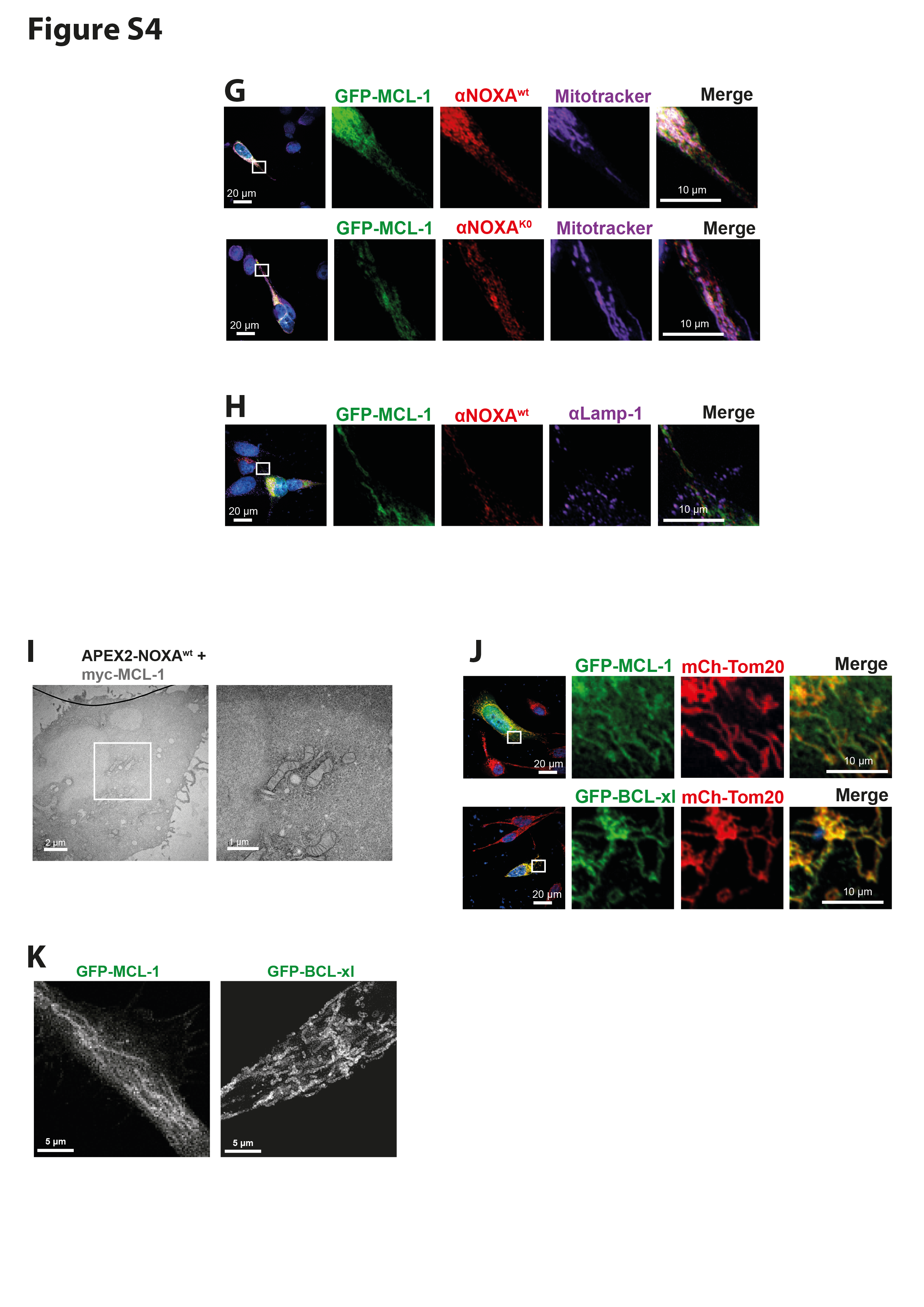

Supplement: Supplementary file 6 — Supplementary Figure S4-2 [file 41419_2020_2923_MOESM6_ESM.tif]

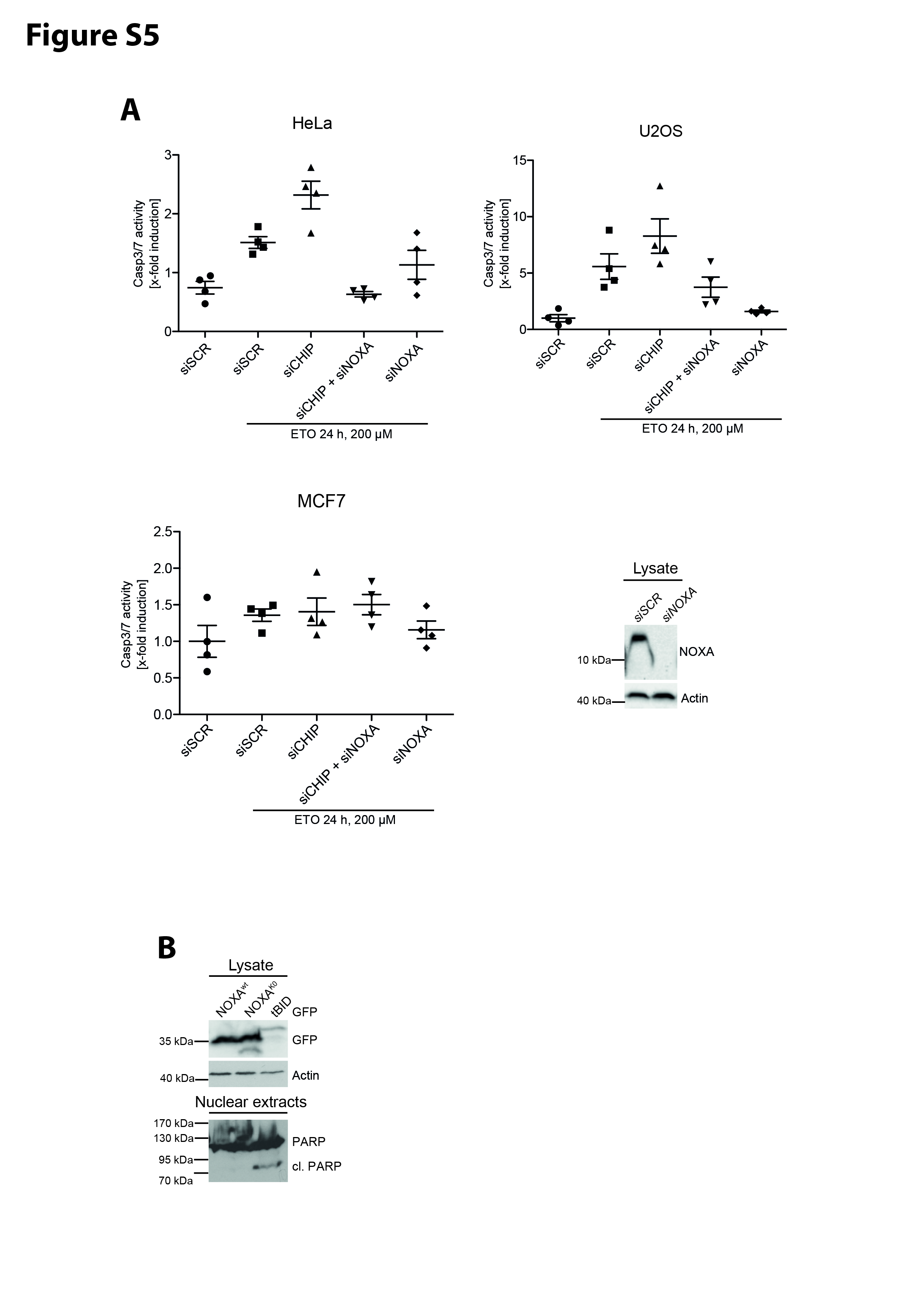

Supplement: Supplementary file 7 — Supplementary Figure S5 [file 41419_2020_2923_MOESM7_ESM.tif]
