# Supplementary material for: CHIP ubiquitylates NOXA and induces its lysosomal degradation in response to DNA damage
Source: Cell Death Dis. 2020 Sep 10;11(9):740. doi: 10.1038/s41419-020-02923-x (PMC7484759; doi:10.1038/s41419-020-02923-x)
Supplement: Supplementary file 8 — Supplementary Figure Legends [file 41419_2020_2923_MOESM8_ESM.docx]

**Fig. S1 DNA damage but not apoptosis induces ubiquitylation of NOXA.** (**A**) Representative measurement of qPCR analysis of HeLa cells treated with indicated DOX concentrations (left panel, n = 6 independent biological samples) or with 1 µM DOX for indicated time points (right panel, n = 4 independent biological samples) to measure *NOXA* mRNA fold induction compared to *Actin* as house-keeping gene. Data represent mean ± SEM. (**B**) Cell death analysis of HCT (upper panel) and HeLa cells (lower panel) treated with 2 µM DOX, 0.25 µM STS or 2 µM DOX with 20 µM zVAD. Cell death was measured by SYTOX Green uptake *via* IncuCyte and calculated as fold induction compared to untreated control. Western blot analysis of cell lysate (Input) and (**C**) Strep-PD of HEK293T cells transfected with Strep-Ub^wt^ for 36 h and treated with 2 µM DOX for indicated time points. (**D**) Strep-PD of HeLa cells transfected with Strep-Ub^wt^ for 36 h and treated with ETO for indicated concentrations. (**E**) Strep-PD of HeLa cells transfected with Strep-Ub^wt^ for 36 h and treated with STS for indicated concentrations. (**F**) Strep-PD of HEK293T cells transfected with Strep-Ub^wt^ and *siSCR* or siRNAs #1 and #2 targeting *RBX1* as indicated for 48 h. All samples were treated with 1 µM DOX for 16 h prior to lysis. (**G**) Representative measurement of qPCR analysis of HeLa cells treated with DOX for indicated time points to measure *CHIP* mRNA fold induction compared to *Actin* as house-keeping gene. Data represent mean ± SEM. n = 4 independent biological samples. ns p> 0.05, * p≤ 0.05, ** p≤ 0.01, *** p≤ 0.001. Asterisk (*) indicates non-specific band. Actin served as loading control in all experiments. NT: not transfected. DOX: Doxorubicin. ETO: Etoposide. STS: Staurosporine. zVAD: zVAD-fmk. All experiments are representatives of at least three independent experiments.

**Fig. S2 CHIP ubiquitylates NOXA.** Cell-free ubiquitylation assay of (**A**) a series of recombinant E2 enzymes and the recombinant proteins flag-NOXA and CHIP. (**B**) Recombinant protein flag-NOXA was incubated with a subset of different E3 ligases as indicated. Western blot analysis of cell lysate (Input) and (**C**) Strep-PD of HeLa cells transfected with Strep-Ub chain-assembly mutants (Kxonly) as indicated for 36 h and treated with 50 nM BORTE for 16 h, (**D**) Strep-PD of HeLa cells transfected with Strep-Ub and NOXA lysine mutants (Kxonly) as indicated for 36 h, (**E**) Strep-PD of HeLa cells transfected with Strep-Ub^wt^ and NOXA lysine mutants (KxR) as indicated for 36 h. (**F**) Western blot analysis of cell lysate (Input) and Strep-PD of HeLa cells transfected with Strep-Ub^wt^ and NOXA lysine mutants (KxxxR) as indicated for 36 h. (**G**) Cell-free ubiquitylation assay of recombinant CHIP and flag-NOXA lysine mutants (KxxxR) as indicated. Actin served as loading control. NT: not transfected. BORTE: Bortezomib. All experiments are representatives of at least three independent experiments.

**Fig. S3 NOXA co-localizes with lysosomes.** (**A**) Confocal microscopy of HeLa cells transfected with untagged NOXA^wt^ for 16 h and stained with NOXA antibody (568 nm) and Mitotracker (633 nm). (**B**) EM of HeLa cells transfected with APEX2-NOXA^wt^ for 16 h. (**C**) Confocal microscopy of HeLa cells transfected with mCh-NOXA^wt^ for 16 h and stained with Rab5 antibody (488 nm), Rab7 antibody (488 nm), Rab9 antibody (488 nm), EEA1 antibody (488 nm), Syntaxin6 antibody (488 nm), Cop1β antibody (488 nm), Vamp3 antibody (488 nm), APPL antibody (488 nm), GopC antibody (488 nm), Sec23 antibody (488 nm). (**D**) Confocal microscopy of HeLa cells transfected for 16 h with untagged NOXA^wt^ and stained with NOXA antibody (568 nm) and Lamp-1 antibody (633 nm). (**E**) Western blot analysis of cell lysate (Input) and GFP-IP of HeLa cells transfected with HA-NOXA^wt^ and GFP-fused proteins as indicated for 36 h. Actin served as loading control. (**F**) Confocal microscopy of HeLa cells transfected for 16 h with untagged NOXA^K0^ and stained with NOXA antibody (568 nm) and Lamp-1 antibody (633 nm) (upper panel), mCherry-NOXA^K0^ and GFP-Rab7 (middle panel), GFP-NOXA^K0^ and stained with Tom20 antibody (568nm) (lower panel). Nuclei were counterstained with DAPI in all confocal microscopic experiments. All experiments are representatives of at least three independent experiments.

**Fig. S4 MCL-1 binding is crucial for mitochondrial association of NOXA.** Western blot analysis of cell lysate (Input) and (**A**) Strep-PD of HeLa cells transfected with Strep-Ub^wt^, HA-NOXA and increasing amounts of myc-MCL-1 as indicated for 36 h and treated with 1 µM DOX for 16 h. (**B**) HA-IP of HeLa cells transfected with HA-NOXA^wt^ and increasing amounts of myc-BCL-xl as indicated for 36 h. (**C**) Strep-PD of HeLa cells transfected with Strep-Ub^wt^, HA-NOXA^wt^ and increasing amounts of myc-BCL-xl as indicated for 36 h. (**D**) HA-IP of HeLa cells transfected with HA-NOXA^wt^ or HA-NOXA^3E^ and increasing amounts of myc-MCL-1 as indicated for 36 h. (**E**) Cell-free ubiquitylation assay of recombinant protein MCL-1 by CHIP. (**F**) Western blot analysis of cell lysate (Input) and HA-IP of HeLa cells transfected with HA-NOXA^wt^ and *siSCR* or two independent siRNAs targeting *MCL-1* (#1, #2) for 48 h. Confocal microscopy of HeLa cells transfected with untagged NOXA^wt^ or NOXA^K0^ (568 nm) with GFP-MCL-1 for 16h and (**G**) mitochondria were visualized by mitotracker (633 nm), (**H**) staining with Lamp-1 antibody (633 nm). (**I**) Electron microscopy (EM) of HeLa cells transfected with APEX2-NOXA and myc-MCL-1 for 16 h. (**J**) Confocal microscopy of HeLa cells transfected with GFP-MCL-1 (upper panel) or GFP-BCL-xl (lower panel) with mCh-Tom20 for 16 h. (**K**) STED microscopy of HeLa cells transfected with GFP-MCL-1 or GFP-BCL-xl for 16 h. Nuclei were counterstained with DAPI in all confocal microscopic experiments. Actin served as loading control. NT: not transfected. DOX: Doxorubicin. mCh: mCherry. All experiments are representatives of at least three independent experiments.

**Fig. S5 Control of NOXA by CHIP serves as a cellular safeguard system.** (**A**) IncuCyte analysis of activated Caspase 3/7 in HeLa, U2OS and MCF7 cell lines transfected with respective siRNAs for 48 h and stimulated with ETO as indicated (upper panels and lower left panel). Western blot analysis of lysates of HeLa cells transfected with respective siRNAs for 48 h (lower right panel). (**B**) Western blot analysis of lysates of HeLa cells transfected with GFP-NOXA^wt^, GFP-NOXA^K0^ and GFP-tBID, respectively, for 20 h. Actin served as loading control. ETO: Etoposide. All experiments are representatives of at least three independent experiments.
