# Supplementary material for: CHIP ubiquitylates NOXA and induces its lysosomal degradation in response to DNA damage
Source: Cell Death Dis. 2020 Sep 10;11(9):740. doi: 10.1038/s41419-020-02923-x (PMC7484759; doi:10.1038/s41419-020-02923-x)
Supplement: Supplementary file 9 — Supplementary Table 1 [file 41419_2020_2923_MOESM9_ESM.docx]

**Tables**

**Supplementary Tab. 1 Putative binding partners of NOXA** MS analysis of NOXA-IP crosslinked with glutaraldehyde (GA) and treated with 1 µM DOX for 16 h. Significant binders were defined as proteins not present in the IgG control (CTRL) determined by total peptide count (Peptides) and label-free quantification (LFQ) normalized intensity (log2 transformed). NOXA, CHIP and RBX1 are labeled yellow.

## Table MS/LS significant binders

| **LFQ intensity** | | | **Peptides** | | |  |
| --- | --- | --- | --- | --- | --- | --- |
| **CTRL** | **GA** | **DOX + GA** | **CTRL** | **GA** | **DOX + GA** | **Gene name** |
| NaN | 27,22729 | NaN | 0 | 3 | 2 | PRDX6 |
| NaN | 25,85736 | NaN | 0 | 2 | 1 | APEX1 |
| NaN | 25,47271 | NaN | 0 | 3 | 0 | PRKCSH |
| NaN | 25,28933 | NaN | 0 | 1 | 0 | NUDT21 |
| NaN | 25,24467 | NaN | 0 | 2 | 2 | CKAP4 |
| NaN | 24,72573 | NaN | 0 | 1 | 0 | ANP32E |
| NaN | 24,62053 | NaN | 0 | 2 | 0 | RIOK1 |
| NaN | 24,28008 | NaN | 0 | 2 | 1 | SLC7A1 |
| NaN | 24,23615 | NaN | 0 | 2 | 0 | STMN2 |
| NaN | 23,98824 | NaN | 0 | 1 | 0 | DDT;DDTL |
| NaN | 23,39244 | NaN | 0 | 1 | 0 | P4HB |
| NaN | 22,55448 | NaN | 0 | 1 | 0 | HYPK |
| NaN | 22,43695 | NaN | 0 | 1 | 0 | PSMC5 |
| NaN | 33,73298 | 34,87179 | 0 | 6 | 6 | PMAIP1 |
| NaN | 30,57975 | 31,33825 | 0 | 17 | 30 | ATP1A1 |
| NaN | 31,01418 | 30,99678 | 0 | 7 | 8 | SNRNP27 |
| NaN | 30,59495 | 30,8282 | 0 | 20 | 22 | EIF5B |
| NaN | 29,28747 | 29,66959 | 0 | 8 | 11 | SLC3A2 |
| NaN | 29,76169 | 29,61121 | 0 | 5 | 5 | BTF3 |
| NaN | 28,92553 | 29,58348 | 0 | 4 | 5 | BSG |
| NaN | 28,72381 | 29,36915 | 0 | 8 | 12 | TFRC |
| NaN | 28,82271 | 29,2138 | 0 | 4 | 7 | DDOST |
| NaN | 28,34617 | 28,85766 | 0 | 4 | 6 | ATP1B3 |
| NaN | 28,04781 | 28,81011 | 0 | 2 | 5 | SLC16A1 |
| NaN | 27,75379 | 28,69102 | 0 | 3 | 7 | GNAI2;GNAI1 |
| NaN | 28,02691 | 28,54749 | 0 | 3 | 3 | LRRC59 |
| NaN | 27,28868 | 28,23169 | 0 | 3 | 4 | RAB7A |
| NaN | NaN | 28,14351 | 0 | 0 | 3 | MT-CO2 |
| NaN | 27,65089 | 28,11481 | 0 | 2 | 7 | RPN2 |
| NaN | 28,11857 | 28,00871 | 0 | 4 | 3 | BTF3L4 |
| NaN | 27,88233 | 27,92428 | 0 | 5 | 5 | GDI2 |
| NaN | 27,81861 | 27,91239 | 0 | 3 | 5 | TBC1D10B |
| NaN | 27,40178 | 27,90805 | 0 | 3 | 2 | RAB1B;RAB1A;RAB1C |
| NaN | NaN | 27,80409 | 0 | 1 | 5 | AK2 |
| NaN | 27,14365 | 27,78631 | 0 | 3 | 3 | RAP1A;RAP1B |
| NaN | 25,73149 | 27,78288 | 0 | 3 | 7 | RHOA |
| NaN | NaN | 27,78232 | 0 | 2 | 3 | GNB2;GNB4 |
| NaN | 27,31984 | 27,76642 | 0 | 3 | 8 | BCAP31 |
| NaN | NaN | 27,57218 | 0 | 1 | 2 | RAB6A;RAB6B;RAB39A |
| NaN | NaN | 27,48454 | 0 | 0 | 1 | COX7A2 |
| NaN | NaN | 27,47977 | 0 | 1 | 3 | STX12 |
| NaN | 27,56827 | 27,46155 | 0 | 3 | 3 | HMGN1 |
| NaN | NaN | 27,4475 | 0 | 0 | 3 | RALA |
| NaN | 26,58849 | 27,33594 | 0 | 2 | 4 | RAB5C;RAB5B |
| NaN | NaN | 27,32877 | 0 | 0 | 3 | DSG2 |
| NaN | 27,39893 | 27,31025 | 0 | 4 | 5 | CCDC124 |
| NaN | 27,142 | 27,30496 | 0 | 2 | 3 | GNB1 |
| NaN | NaN | 27,23087 | 0 | 1 | 4 | RAB11A;RAB11B |
| NaN | 27,14014 | 27,2141 | 0 | 3 | 4 | ZMPSTE24 |
| NaN | NaN | 27,17807 | 0 | 0 | 2 | COX5A |
| NaN | 26,88449 | 27,17427 | 0 | 3 | 3 | RAB10 |
| NaN | NaN | 27,11486 | 0 | 1 | 2 | VAMP3;VAMP2 |
| NaN | NaN | 27,08192 | 0 | 2 | 5 | ATP2B1 |
| NaN | NaN | 27,04124 | 0 | 0 | 5 | RAB14 |
| NaN | NaN | 27,03128 | 0 | 1 | 1 | IGHG2 |
| NaN | 27,10039 | 26,92863 | 0 | 2 | 3 | DNAJC5 |
| NaN | NaN | 26,89331 | 0 | 2 | 3 | COX4I1 |
| NaN | NaN | 26,86467 | 0 | 0 | 2 | PHB |
| NaN | NaN | 26,82457 | 0 | 1 | 4 | ANXA2;ANXA2P2 |
| NaN | 26,40121 | 26,78681 | 0 | 2 | 2 | CD99 |
| NaN | 26,14209 | 26,78469 | 0 | 2 | 2 | TMED10 |
| NaN | NaN | 26,73686 | 0 | 1 | 1 | HACD3 |
| NaN | NaN | 26,69896 | 0 | 0 | 1 | KRAS;HRAS;NRAS |
| NaN | NaN | 26,56564 | 0 | 2 | 4 | GNAS |
| NaN | NaN | 26,56538 | 0 | 0 | 2 | ATP1B1 |
| NaN | NaN | 26,39375 | 0 | 1 | 3 | STT3B |
| NaN | NaN | 26,3562 | 0 | 1 | 3 | LMAN1 |
| NaN | 26,18527 | 26,34303 | 0 | 2 | 2 | TPD52L2 |
| NaN | NaN | 26,30603 | 0 | 2 | 4 | ARF4 |
| NaN | NaN | 26,27154 | 0 | 1 | 4 | VPS35 |
| NaN | 26,52868 | 26,26642 | 0 | 2 | 2 | ANP32B |
| NaN | NaN | 26,18939 | 0 | 0 | 2 | ATP11C |
| NaN | NaN | 26,14703 | 0 | 1 | 2 | PGRMC1 |
| NaN | NaN | 26,01527 | 0 | 0 | 2 | EZR;RDX |
| NaN | NaN | 25,93123 | 0 | 0 | 2 | STUB1 |
| NaN | 26,06919 | 25,91137 | 0 | 3 | 2 | CSE1L |
| NaN | NaN | 25,89231 | 0 | 1 | 2 | KLHDC4;DKFZp434G0522 |
| NaN | NaN | 25,88159 | 0 | 2 | 2 | VAPA |
| NaN | 25,81274 | 25,85529 | 0 | 2 | 2 | PDIA6 |
| NaN | NaN | 25,83685 | 0 | 1 | 1 | HLA-A;HLA-C;HLA-H; HLA-B |
| NaN | NaN | 25,81744 | 0 | 0 | 2 | PEBP1 |
| NaN | NaN | 25,79731 | 0 | 0 | 2 | VAMP7 |
| NaN | NaN | 25,76891 | 0 | 1 | 1 | SLC12A2 |
| NaN | NaN | 25,74771 | 0 | 0 | 2 | SEC22B |
| NaN | NaN | 25,7473 | 0 | 0 | 4 | EPB41L2 |
| NaN | 25,86486 | 25,73898 | 0 | 2 | 2 | CDC42;RHOQ;RAC2; RHOJ;RHOG;RAC3 |
| NaN | 25,6421 | 25,72918 | 0 | 2 | 3 | STT3A |
| NaN | NaN | 25,71487 | 0 | 0 | 1 | NEDD8;NEDD8-MDP1 |
| NaN | NaN | 25,68835 | 0 | 1 | 2 | RAB35 |
| NaN | 26,18082 | 25,65706 | 0 | 2 | 2 | ERO1L |
| NaN | NaN | 25,649 | 0 | 1 | 1 | DCD |
| NaN | NaN | 25,64058 | 0 | 1 | 2 | ATP2A2;ATP2A1 |
| NaN | NaN | 25,58867 | 0 | 1 | 1 | RAB2A |
| NaN | NaN | 25,36162 | 0 | 0 | 3 | DNAJC7 |
| NaN | NaN | 25,29835 | 0 | 0 | 2 | RRAS2 |
| NaN | NaN | 25,18922 | 0 | 1 | 2 | TMED2 |
| NaN | NaN | 25,18056 | 0 | 0 | 1 | COX7C |
| NaN | NaN | 25,16779 | 0 | 1 | 2 | STX7 |
| NaN | NaN | 25,13884 | 0 | 1 | 1 | CKMT1A;CKMT2;CKMT1B |
| NaN | NaN | 25,11223 | 0 | 1 | 3 | RAB5A |
| NaN | NaN | 25,09937 | 0 | 1 | 2 | EPB41L3 |
| NaN | NaN | 25,09112 | 0 | 0 | 1 | AGTRAP |
| NaN | NaN | 25,08982 | 0 | 0 | 1 | MTPN |
| NaN | NaN | 25,08712 | 0 | 0 | 2 | ACBD3 |
| NaN | NaN | 25,08241 | 0 | 1 | 1 | SLC7A5 |
| NaN | NaN | 25,06085 | 0 | 1 | 1 | ETFA |
| NaN | NaN | 25,02968 | 0 | 1 | 2 | ERLIN2;ERLIN1 |
| NaN | NaN | 25,00131 | 0 | 0 | 2 | CNP |
| NaN | NaN | 24,97561 | 0 | 1 | 1 | FKBP3 |
| NaN | 24,79734 | 24,93049 | 0 | 2 | 2 | SRPRB |
| NaN | NaN | 24,86839 | 0 | 2 | 5 | GNAI3 |
| NaN | NaN | 24,85158 | 0 | 0 | 2 | KPNA2 |
| NaN | NaN | 24,79769 | 0 | 0 | 2 | STOML2 |
| NaN | NaN | 24,79169 | 0 | 1 | 1 | DNAJC8 |
| NaN | NaN | 24,69785 | 0 | 1 | 2 | GNG12 |
| NaN | NaN | 24,69471 | 0 | 1 | 5 | ATP2B4 |
| NaN | NaN | 24,68891 | 0 | 0 | 2 | PITPNB |
| NaN | NaN | 24,62433 | 0 | 1 | 2 | SLC1A3 |
| NaN | NaN | 24,59508 | 0 | 1 | 1 | ZRANB2 |
| NaN | NaN | 24,54616 | 0 | 0 | 2 | RAP2C;RAP2A;RAP2B |
| NaN | NaN | 24,4954 | 0 | 0 | 1 | NDUFB11 |
| NaN | NaN | 24,45532 | 0 | 0 | 1 | SNX2 |
| NaN | NaN | 24,40944 | 0 | 1 | 1 | TALDO1 |
| NaN | NaN | 24,40503 | 0 | 0 | 2 | STX4 |
| NaN | NaN | 24,37968 | 0 | 0 | 1 | HLA-B |
| NaN | NaN | 24,36346 | 0 | 1 | 1 | FKBP12-Exin;FKBP1A |
| NaN | NaN | 24,32636 | 0 | 1 | 2 | TMED9 |
| NaN | NaN | 24,31403 | 0 | 1 | 1 | COX17 |
| NaN | NaN | 24,31264 | 0 | 0 | 1 | JUP |
| NaN | NaN | 24,3048 | 0 | 0 | 1 | MDH1 |
| NaN | NaN | 24,24837 | 0 | 0 | 1 | COPB2 |
| NaN | NaN | 24,22678 | 0 | 0 | 1 | HIGD1A |
| NaN | NaN | 24,2126 | 0 | 0 | 2 | GNA14;GNAQ;GNA11; GNA12;GNAZ;GNA13 |
| NaN | NaN | 24,19603 | 0 | 1 | 1 | TMX1 |
| NaN | NaN | 24,14872 | 0 | 1 | 1 | PPA1 |
| NaN | NaN | 24,07496 | 0 | 0 | 1 | SCAMP3 |
| NaN | NaN | 24,05656 | 0 | 1 | 2 | HMGN4 |
| NaN | NaN | 24,05308 | 0 | 0 | 1 | KLRG2 |
| NaN | NaN | 24,04743 | 0 | 1 | 1 | TMEM30A |
| NaN | NaN | 23,99878 | 0 | 0 | 1 | SNAP23 |
| NaN | NaN | 23,98416 | 0 | 1 | 1 | SLC1A5 |
| NaN | NaN | 23,96217 | 0 | 1 | 1 | COA6 |
| NaN | NaN | 23,94575 | 0 | 1 | 2 | PRDX4 |
| NaN | NaN | 23,9453 | 0 | 0 | 1 | ALDH3A2 |
| NaN | NaN | 23,89624 | 0 | 0 | 1 | CPNE3 |
| NaN | NaN | 23,87568 | 0 | 0 | 1 | ZNF593 |
| NaN | NaN | 23,8641 | 0 | 0 | 1 | RP2 |
| NaN | NaN | 23,8042 | 0 | 0 | 1 | SFXN1 |
| NaN | NaN | 23,80302 | 0 | 0 | 2 | IGF2R |
| NaN | NaN | 23,79709 | 0 | 0 | 1 | STXBP3 |
| NaN | NaN | 23,72557 | 0 | 0 | 1 | ESYT1 |
| NaN | NaN | 23,64823 | 0 | 1 | 1 | ARL8B |
| NaN | NaN | 23,53991 | 0 | 0 | 1 | ATP6V1G1 |
| NaN | NaN | 23,39297 | 0 | 0 | 1 | TBL2 |
| NaN | NaN | 23,22394 | 0 | 1 | 1 | UBE2N;UBE2NL |
| NaN | NaN | 23,1327 | 0 | 0 | 1 | TXNDC5 |
| NaN | NaN | 23,0355 | 0 | 0 | 1 | RBX1 |
| NaN | NaN | 22,95363 | 0 | 1 | 1 | YARS |
| NaN | NaN | 22,82024 | 0 | 0 | 1 | RAB9A |
